# Dual-action peptide KWH_2_ protects against *Salmonella choleraesuis* diarrhea in weaned piglets by enhancing intestinal barrier integrity and modulating GSK-3β/Myc signaling

**DOI:** 10.1186/s13567-025-01682-x

**Published:** 2026-03-17

**Authors:** Yunhui Zhu, Kaikai Lv, Ziye Tian, Yueyang Han, Zhongpeng Bi, Na Dong

**Affiliations:** 1 The Laboratory of Molecular Nutrient and Immunity, College of Animal Science and Technology, Northeast Agricultural University, Harbin, China; 2 College of Animal Science & Technology, Henan University of Animal Husbandry and Economy, Henan, China

**Keywords:** Immunoregulatory peptide, GSK-3β/Myc, *Salmonella choleraesuis*, glucose homeostasis, intestinal barrier

## Abstract

Bioactive peptides with dual antimicrobial and immune-regulatory functions offer promising strategies for managing infections and immune dysregulation in livestock. KWH_2_, a novel peptide demonstrating potent antimicrobial activity, holds translational potential for enteric disease control. This study evaluated KWH_2_’s therapeutic efficacy in weaned piglets challenged with *Salmonella choleraesuis*—a clinically relevant model of intestinal infection and immunity. KWH_2_ supplementation significantly improved clinical outcomes during infection, including restoring growth performance, reducing diarrhea severity, and enhancing intestinal barrier integrity. Transcriptomic analysis revealed KWH_2_’s modulation of key pathways in intestinal immunity, inflammation, and cellular stress responses. Mechanistically, KWH_2_ targeted the *GSK-3β* signaling axis, suppressing inflammatory activation and promoting epithelial repair through GSK-3β phosphorylation. These results establish KWH_2_ as a dual-function therapeutic candidate that enhances intestinal resilience against infection by integrating antimicrobial activity with host-directed immunometabolic regulation. Its efficacy in a production-relevant porcine model supports KWH_2_’s potential for developing veterinary therapeutics targeting enteric pathogens.

**Supplementary Information:**

The online version contains supplementary material available at 10.1186/s13567-025-01682-x.

## Introduction

The intestinal tract serves as both a physical barrier and a dynamic immune interface, orchestrating host defense against pathogens while maintaining metabolic and immunological homeostasis [1]. Postweaning piglets are highly susceptible to enteropathogens (e.g., *Salmonella* spp.) [2, 3]. This susceptibility stems from their immature immune system and is promoted by intestinal dysbiosis after diet change (from milk-based to grain-based feed) and the stress associated with new environments. Postweaning diarrhea exerts long-term consequences on the growth performance and systemic health of piglets. Emerging therapeutic strategies targeting immune‒metabolic crosstalk and pathogen-induced immune dysregulation are urgently needed to address this multifactorial challenge [4].

Antimicrobial peptides (AMPs) represent a promising class of immunopharmaceuticals owing to their dual antimicrobial efficacy and capacity to modulate host immunity [5–7]. We previously reported a bioactive peptide, KWH_2_, which exhibits potent bactericidal activity against enteric pathogens and enhances epithelial resilience by promoting intestinal cell viability [8]. The immunomodulatory properties of KWH_2_ position it as a candidate for prophylactic intervention during the vulnerable postweaning period. This aligns with the paradigm shift toward early immunotherapeutic strategies, as late-stage treatments often fail to reverse established mucosal damage.

Here, we investigated the prophylactic potential of KWH_2_ in a *Salmonella*-challenged weaning pig model, focusing on its ability to increase intestinal barrier integrity and resolve infection-driven immune dysregulation. *Salmonella choleraesuis* was selected for this challenge. Among *Salmonella enterica* serovars in pigs, *Salmonella typhimurium* is the most common serotype, whose typical clinical signs includes diarrhea and vomiting. However, the clinical manifestations of *Salmonella typhimurium* are often variable, ranging from subclinical carriage to gastroenteritis—making it less ideal for evaluating the prophylactic effects of KWH_2_. However, *Salmonella choleraesuis* is more rigorous, and symptoms are more severe and prolonged following infection with *Salmonella choleraesuis*. Thus, *Salmonella choleraesuis* was selected as a pathogenic bacterial model for postweaning diarrhea in this study, though *Salmonella choleraesuis* is not typically associated with postweaning diarrhea in field settings. We propose that KWH_2_ acts through the glycogen synthase kinase-3β (GSK-3β)/*MYC* proto-oncogene (Myc) signaling axis—a pathway central to immune metabolism and epithelial repair—to pre-emptively counteract bacterial pathogenesis. By bridging antimicrobial action with immunoregulatory mechanisms, this study advances the translational potential of peptide-based therapeutics in managing infection-associated mucosal disorders.

## Materials and methods

### Animal ethics

The animal experiments were approved by the Northeast Agricultural University (NEAU) Institutional Animal Care and Use Committee. The treatments complied with the NEAU Animal Welfare Committee Protocol (NEAU-[2013]−9).

### Animals and treatments

A total of 32 weaning pigs (duroc × long white × large white; 7.7 ± 0.68 kg; 28 days of age; all barrows) were recruited. Upon arrival, the pigs were divided into four groups (*n* = 8) balanced by body weight and adaptively fed for 5 days. The diets used were prepared on the basis of the Chinese National Standard *Nutrient requirements of swine* (GB/T 39235-2020). The formula and nutrient levels of the diets are presented in Table 1. The nutrient compositions of the basal diet were determined in accordance with national standards. The crude protein content (*N* × 6.25) was determined via a KDN-16K Kjeldberg nitrogen distiller (Qianjian, China; GB/T 6432–2018). The crude fat content was measured via ether extraction (GBT 6433-2006) with a Soxhlet extractor (JC-ST-06, Benang, China). Crude fiber was assessed via an automatic fiber analyzer (ANKOM TDFi, USA; GBT 6434-2006/ISO 6865 2000). Total calcium was measured via the potassium permanganate method in compliance with GB/T 6436-2018, and total phosphorus was measured via the colorimetric molybdovanadate method (GB/T 6437-2018). Net energy was calculated on the basis of values from Tables of Feed Composition and Nutritive Values in China. Pigs were housed in temperature-controlled pens (25 ± 2 °C), with feed and water ad libitum throughout the experiment. The bioactive peptide KWH_2_, used in this study, was designed by hybridizing a chemotactic motif (MMHWFM) with an AMP sequence (*Ph*HA*hP*H)_n_. KWH_2_ was synthesized via solid-phase synthesis by Sangon Biotech (Shanghai, China) Co., Ltd. The purity of the synthesized peptides was confirmed via reversed-phase high-performance liquid chromatography (RP-HPLC; Additional file 2) with a Shimdze Inertsil octadecylsilane special-phase column (4.6 mm × 250 mm × 5 μm) and matrix-assessed laser desorption/ionization time-of-flight mass spectrometry (MALDI-TOF MS, Linear Scientific, Inc., USA; Additional file 1). The data suggested that the purity of KWH_2_ was greater than 95%. KWH_2_ exhibited an average minimum inhibitory concentration (MIC) against gram-negative bacteria and an MIC = 4 μM against *Salmonella choleraesuis* in vitro. The gastric and intestinal stability of KWH_2_ was examined via HPLC (Additional file 2).

**Table 1:** **Formula and nutrient levels of the basal diet (%, as-dry-matter basis)**

Item	Content	Nutrient level	Content
Corn	52.30	Net energy, MJ/kg	10.88
Soybean meal	17.70	Crude protein^a^	17.10
Puffed corn	10.00	Crude fat^a^	6.51
Extruded soybean	7.00	Crude fiber^a^	2.05
Whey powder	5.00	Calcium^a^	0.63
Soybean oil	1.50	Total phosphorus^a^	0.54
Coconut oil	1.00	Available phosphorus	0.41
Steam-dried fish meal	2.00	Lysine	1.25
Dicalcium phosphate^b^	1.00	Methionine	0.43
Limestone	0.80	Methionine + cystine	0.72
Lysine HCl	0.51	Threonine	0.82
dl-Methionine	0.20	Tryptophan	0.26
l-Threonine	0.20	Valine	0.86
l-Tryptophan	0.10	Isoleucine	0.63
Valine	0.10		
Salt	0.35		
Choline chloride, 60%	0.05		
Mineral premix^c^	0.15		
Vitamin premix^d^	0.04		
Total	100.00		

^a^Measured value. The remaining nutrient levels were calculated as follows:

^b^Mixture of dicalcium phosphate and monocalcium phosphate, containing 16% calcium and 21% phosphorus

^c^Mineral premix provided the following per kilogram of diet: Fe: 80.0 mg; Zn: 100.0 mg; Cu: 12.5 mg; Mn: 8.2 mg; I: 0.15 mg; Se: 0.2 mg

^d^Vitamin premix provided the following per kilogram of diet: vitamin A: 5500 IU; vitamin D3: 1000 IU; vitamin E: 100 IU; vitamin K: 2.0 mg; vitamin B1: 3.8 mg; vitamin B2: 6.2 mg; niacin: 36.0 mg; pantothenic acid: 20.0 mg; vitamin B6: 3.5 mg; biotin: 0.3 mg; folacin: 1.4 mg; vitamin B12: 0.03 mg.

**Figure 1:**
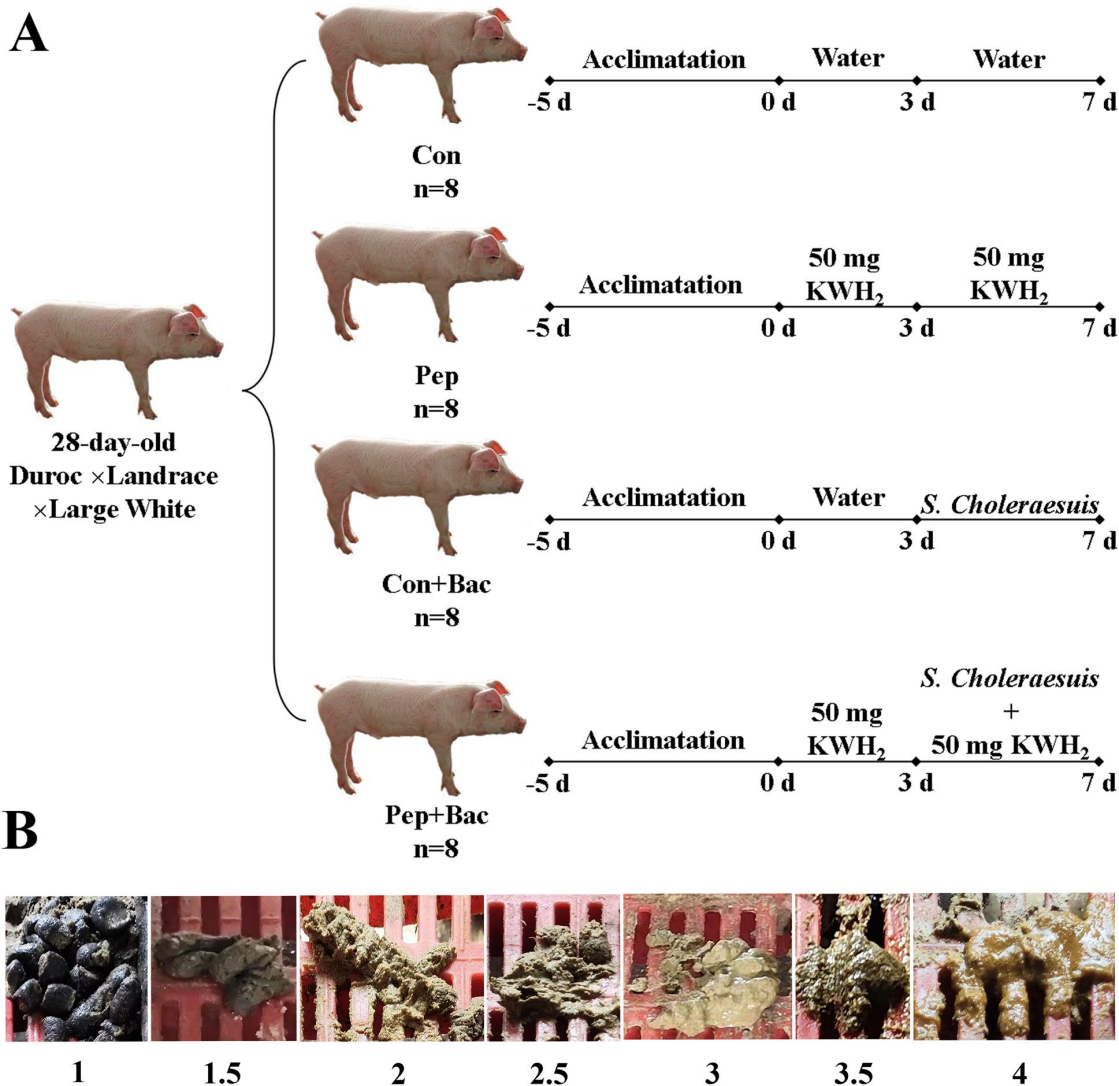
**Schematic diagram of the experimental design and representative photos for feces score.** **A** Schematic diagram of the experimental design. **B** Representative photographs taken from the experimental site for the fecal consistency (FC) scoring system. Value of 1 for firm feces indicates normal status; 2 for pasty feces indicates slight diarrhea; 3 for semi-liquid feces indicates moderate diarrhea; and 4 for liquid and unformed feces indicates severe diarrhea.

As shown in Figure 1A, the treatments lasted for 7 days and included two phases (prevention for 3 days [from day 0 to day 3]; treatment for 4 days [from day 3 to day 7]). The details of the treatments were as follows:

1. Control (Con, no operation for prevention [day 0–3] or treatment [day 3–7]);
2. Peptide only (Pep; 50 mg/day/pig KWH_2_ for prevention and 50 mg/day/pig KWH_2_ for treatment without *Salmonella choleraesuis* infection throughout the experiment);
3. *Salmonella choleraesuis* infection only (Con + Bac; *Salmonella choleraesuis* infection from days 3 to 7 without KWH_2_ throughout the experiment);
4. Peptide + *Salmonella choleraesuis* (Pep + Bac; *Salmonella choleraesuis* infection from days 3 to 7 with KWH_2_ throughout the experiment).

KWH_2_ and *Salmonella choleraesuis* were administered by gavage. For KWH_2_ administration, 50 mg of peptide in 50 mL of distilled water was used; an equal volume of distilled water was used as a control. For *Salmonella choleraesuis* infection, 80 mL of Luria–Bertani (LB) culture medium containing 1 × 10^10^ CFU *Salmonella choleraesuis* was used [9]; an equal volume of culture medium was used as a control. KWH_2_/distilled water was given at 8:00 (30 min after feeding), and *Salmonella choleraesuis*/LB medium was given at 14:00 (6.5 h after feeding).

On days 0, 3, and 7, feed intake and body weight were recorded to calculate the average daily gain (ADG), average daily feed intake (ADFI), and feed-to-gain ratio (F:G) during each phase and overall. The occurrence and severity of diarrhea were evaluated via the fecal consistency score system (Figure 1B): 1 for firm feces, indicating normal status; 2 for pasty feces, indicating slight diarrhea; 3 for semi-liquid feces, indicating moderate diarrhea; and 4 for liquid and unformed feces, indicating severe diarrhea. Fecal consistency was assessed twice daily. Pigs with average daily scores higher than 1 were considered to have diarrhea. Diarrhea rate = (number of diarrheal pigs)/(total number of experimental pigs × experimental time (*d*)). Diarrhea index = total fecal score/total number of experimental pigs.

At the end of the experiment, the pigs were euthanized. Blood samples were collected via the portal vein. Serum was obtained after centrifugation at 3500 *g* and stored at −20 °C until analysis. Tissues for histological staining were preserved in 4% paraformaldehyde; tissues for scanning electron microscope imaging were preserved in 2.5% glutaraldehyde solution. Tissues for morphological analysis were stored at 4 °C, and the remaining collected tissues were immediately snap frozen and stored at −80 °C until analysis.

### Intestinal permeability

Intestinal permeability was assessed on the basis of the serum d-lactic acid (d-Lac) concentration and diamine oxidase (DAO) activity via commercially available enzyme-linked immunosorbent assay (ELISA) kits (Grace Biotechnology, Suzhou, China).

### Intestinal morphology

#### Hematoxylin and eosin (H&E) staining

The preserved tissues were embedded in paraffin and sectioned into slices with a thickness of 5 μm. H&E staining was performed as previously described [10]. Images were acquired with a Nikon B80i microscope (Nikon, Tokyo, Japan).

#### Scanning electron microscopy

The preserved tissues were dehydrated in a graded ethanol series (30%, 50%, 70%, 80%, 90%, 95%, and 100%) for 10 min each, followed by vacuum drying overnight. Dried sections were ion-sputtered and imaged with an S-4800n scanning electron microscope (Hitachi, Japan). The diameter and density of the microvilli (*n* ≥ 60) were determined via Adobe Photoshop 24 (Adobe Inc., USA).

### Jejunum transcriptomic analysis

Total RNA extraction, mRNA library construction, and sequencing were performed at Lianchuan Bio (Hangzhou, China). Total RNA was extracted via TRIzol reagent (Thermo Fisher, 15596018), purified via Dynabeads Oligo (Thermo Fisher, CA, USA), fragmented into short fragments via divalent cations (Magnesium RNA Fragment Module, NEB, e6150, USA), and then reverse-transcribed to cDNA via SuperScript^™^ II Reverse Transcriptase (1896649, Invitrogen, USA). U-labeled second-strand DNAs were synthesized with *Escherichia coli* DNA polymerase I (NEB, cat. no. m0209, USA), RNase H (NEB, cat. no. m0297, USA), and dUTP Solution (Thermo Fisher, cat. no. R0133, USA).

A cDNA library was constructed by sequencing the transcriptome via the Illumina paired-end RNA sequencing (RNA-seq) approach (Illumina NovaSeq^™^6000 sequence platform). The reads obtained were filtered by Cutadapt [11]. Reads containing adapters, polyA and polyG, more than 5% unknown nucleotides, and more than 20% low-quality bases were removed. The quality of the remaining bases was verified via FastQC [12]. The HISAT2 [13] package was used to map the clean reads to the reference genome. The mapped reads were then assembled via the StringTie [14] and reconstructed into a comprehensive transcriptome via gffcompare software [15]. The expression levels were estimated by calculating the fragment per kilobase of transcript per million mapped reads (FPKM) value via StringTie and Ballgown [16]. The correlation analysis of replicas and principal component analysis were performed via R [17]. Differentially expressed genes were analyzed via DESeq2 software. Genes whose false discovery rate (FDR) was less than 0.05 and whose absolute fold change was ≥ 2 were considered differentially expressed genes. The differentially expressed genes were then subjected to enrichment analysis of Gene Ontology [18] functions and pathway enrichment analysis [19].

### Western blot

Total protein was extracted with radioimmunoprecipitation assay (RIPA) lysis buffer (Beyotime, China). The concentration of total protein was determined with a bicinchoninic acid (BCA) assay protein assay kit (Beyotime, China). Proteins were separated via 10% sodium dodecyl sulfate‒polyacrylamide gel electrophoresis (SDS‒PAGE) and then transferred to a polyvinylidene fluoride (PVDF) membrane. The primary antibodies used were as follows: GSK-3β, p-GSK-3β, interleukin-6 (IL-6), interleukin-10 (IL-10), Myc, antigen Kiel 67 (Ki-67), and cleaved caspase-3 (1:2000; Servicebio Technology Co., Ltd., Wuhan, China). The protein bands were visualized with an enhanced chemiluminescence (ECL) detection kit (Beyotime, China) on a ChemiDoc Touch Imaging System (Bio-Rad, USA). The visualized bands were analyzed via ImageJ software (NIH, USA). Each blot represents at least three independent samples.

### Statistical analysis

IBM SPSS 27.0 (Chicago, IL, USA) was used for the statistical analysis, and GraphPad Prism 9 (San Diego, CA, USA) was used to visualize the data. Data on piglet growth performance (ADG, ADFI, and F:G), severity of diarrhea (diarrhea rate and index), and intestinal permeability (DAO and _D_-Lac) were analyzed on the basis of individuals as a unit. H&E staining data were based on villi and crypts as units. Standard error of the mean (SEM) data were based on microvilli as a unit. One-way analysis of variance and Ducan’s multiple range test for pairwise comparisons were employed to determine the effects of KWH_2_ on growth performance (ADG, ADFI, and F:G) and intestinal morphology (villus height, crypt depth, ratio of villus height/crypt, density and diameter of microvilli, and intestinal permeability [DAO and d-Lac]). A nonparametric Kruskal‒Wallis test with Bonferroni-corrected Mann–Whitney *U* test as post hoc analysis was employed to determine the effects of KWH_2_ on the occurrence and severity of diarrhea (diarrhea rate and index). The data are expressed as the means with standard errors of the means (SEMs). A *P*-value less than or equal to 0.05 was considered statistically significant. A *P*-value between 0.05 and 0.10 was considered a trend.

## Results

### KWH_2_ alleviated the effects of *Salmonella choleraesuis* on the growth performance, occurrence and severity of diarrhea, and intestinal integrity of piglets

We first investigated the effects of KWH_2_ on growth performance (Tables 2 and 3). Under basal conditions, KWH_2_ had no effect on the final body weight (day 7), ADG, ADFI, or F:G overall, but KWH_2_ tended to increase the ADFI from day 3 to day 7 (*P* < 0.10). *Salmonella choleraesuis* (1) decreased final body weight (day 7; *P* < 0.001), (2) decreased ADG and ADFI during the treatment phase (*P* < 0.001) and overall (*P* < 0.001 for ADG and *P* = 0.027 for ADFI), (3) increased F:G during the treatment phase (*P* = 0.005), and (4) tended to increase F:G overall (*P* = 0.096). KWH_2_ abolished the deleterious effects of *Salmonella choleraesuis* on (1) final body weight (day 7; *P* < 0.001), (2) ADG during the treatment phase and overall (*P* < 0.001), and (3) ADFI (*P* < 0.001) and F:G during the treatment phase (*P* = 0.005). KWH_2_ tended to reverse the negative effect of *Salmonella choleraesuis* on overall F:G (*P* = 0.096).

**Table 2:** **KWH**_2_** alleviated the effects of *Salmonella choleraesuis* on body weight and average daily gain (ADG) of weaning piglets**

Item	Treatment				*P*-value
	Con	Pep	Con + Bac	Pep + Bac	
Body weight, kg					
Day −5	7.61 ± 0.209	7.68 ± 0.225	7.50 ± 0.214	7.88 ± 0.323	0.751
Day 0	9.26 ± 0.385	9.39 ± 0.363	9.14 ± 0.289	9.85 ± 0.146	0.379
Day 3	11.47 ± 0.451	10.88 ± 0.436	10.74 ± 0.319	11.26 ± 0.296	0.511
Day 7	14.41 ± 0.290^a^	14.49 ± 0.383^a^	12.11 ± 0.408^b^	14.63 ± 0.270^a^	< 0.001
ADG, g/d					
Acclimated (day −5 to day 0)	361.4 ± 54.00	337.5 ± 36.24	318.8 ± 36.52	390.0 ± 54.64	0.708
Prevention (day 0 to day 3)	654.0 ± 34.44	598.7 ± 48.16	617.1 ± 84.76	571.4 ± 91.30	0.706
Treatment (day 3 to day 7)	740.0 ± 71.71^a^	910.0 ± 83.88^a^	347.5 ± 76.99^b^	862.9 ± 84.51^a^	< 0.001
Overall (day 0 to day 7)	732.9 ± 34.07^a^	708.8 ± 54.92^a^	453.8 ± 52.37^b^	714.3 ± 27.68^a^	< 0.001

ADG: average daily gain. The values are the means ± SEMs (*n* = 8); the mean values within a row with different superscript letters (a and b) are significantly different (*P* < 0.05). The mean values within a row with different superscript letters (e and f) tended to differ (*P* < 0.10). Con: basal diet without treatment, Pep: basal diet + 50 mg/day KWH_2_ per pig, Con + Bac: basal diet + *Salmonella choleraesuis* infection, Pep + Bac: basal diet + 50 mg/day KWH_2_ per pig + *Salmonella choleraesuis* infection.

**Table 3:** **KWH**_**2**_** alleviated the effects of *Salmonella choleraesuis* on average daily feed intake (ADFI) and feed intake/weight gain (F:G) of weaning piglets**

Item	Treatment				*P*-value
	Con	Pep	Con + Bac	Pep + Bac	
ADFI, g/day					
Acclimated (day −5 to day 0)	534.4 ± 74.95	506.4 ± 38.66	490.8 ± 25.37	590.6 ± 63.19	0.524
Prevention (day 0 to day 3)	864.4 ± 74.48	687.9 ± 59.51	779.8 ± 81.88	717.1 ± 65.96	0.283
Treatment (day 3 to day 7)	987.5 ± 68.64^a,f^	1219.1 ± 116.06^a,e^	624.3 ± 48.80^b^	1168.9 ± 82.30^a,ef^	< 0.001
Overall (day 0 to day 7)	987.4 ± 55.74^a^	983.8 ± 68.26^a^	738.3 ± 30.63^b^	843.0 ± 84.89^ab^	0.027
F:G, g/g					
Acclimated (day −5 to day 0)	1.53 ± 0.076	1.54 ± 0.093	1.38 ± 0.095	1.56 ± 0.072	0.498
Prevention (day 0 to day 3)	1.43 ± 0.106	1.43 ± 0.045	1.39 ± 0.098	1.44 ± 0.061	0.967
Treatment (day 3 to day 7)	1.36 ± 0.060^b^	1.34 ± 0.030^b^	1.88 ± 0.229^a^	1.41 ± 0.057^b^	0.005
Overall (day 0 to day 7)	1.39 ± 0.097^f^	1.32 ± 0.103^f^	2.09 ± 0.452^e^	1.34 ± 0.094^f^	0.096

The values are the means ± SEMs (*n* = 8); the mean values within a row with different superscript letters (a and b) are significantly different (*P* < 0.05). The mean values within a row with different superscript letters (e and f) tended to differ (*P* < 0.10). ADFI: average daily feed intake, *F:G* feed to gain ratio, Con: basal diet without treatment, Pep: basal diet + 50 mg/day KWH_2_ per pig, Con + Bac: basal diet + *Salmonella choleraesuis* infection, Pep + Bac: basal diet + 50 mg/day KWH_2_ per pig + *Salmonella choleraesuis* infection.

**Figure 2:**
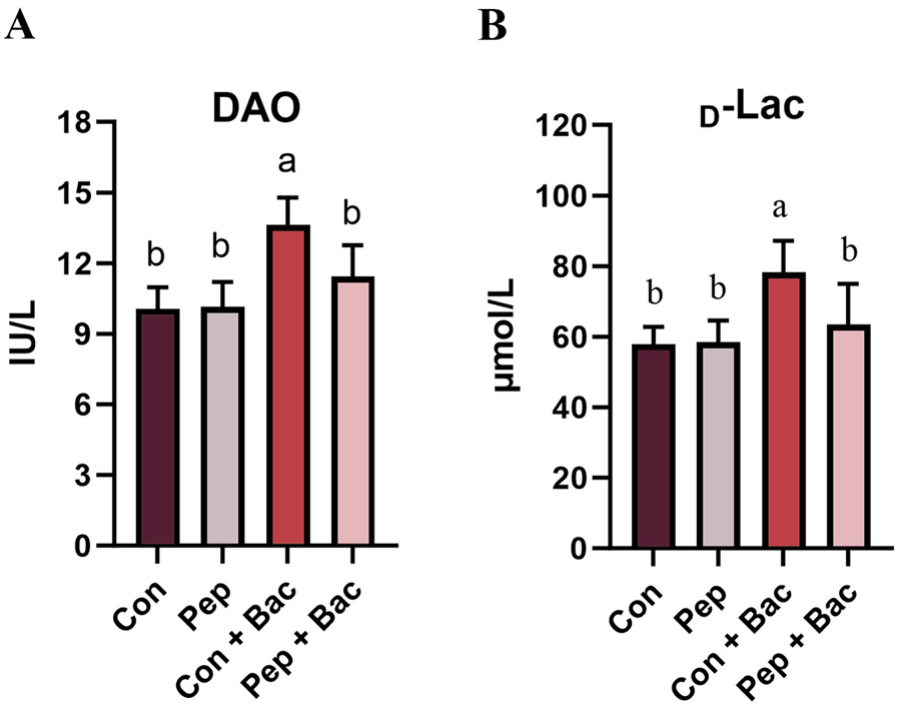
**Effects of KWH**_2_ **on the intestinal permeability of weanling pigs.** **A** Serum DAO activity. **B** The concentration of serum d-Lac. The values are the means ± SEMs (*n* = 8). Columns with different letters are significantly different (*P* < 0.05). DAO: diamine oxidase, _*D*_*-Lac*_D_-lactic acid, Con: basal diet without treatment, Pep: basal diet + 50 mg/day KWH_2_ per pig, Con + Bac: basal diet + *Salmonella choleraesuis* infection, Pep + Bac: basal diet + 50 mg/day KWH_2_ per pig + *Salmonella choleraesuis* infection.

The occurrence and severity of diarrhea were evaluated with the diarrhea rate and diarrhea index (Tables 4 and 5). The effects of *Salmonella choleraesuis* on diarrhea started on day 5, the second day of infection (*P* < 0.05). In general, KWH_2_ reduced the occurrence and attenuated the severity of diarrhea caused by *Salmonella choleraesuis* infection. The intestinal permeability was evaluated with serum DAO and d-Lac (Figure 2). *Salmonella choleraesuis* increased intestinal permeability (*P* < 0.05), whereas the levels of DAO and d-Lac in KWH_2_ and KWH_2_ + *Salmonella choleraesuis* did not differ from those in the control.

**Table 4:** **KWH**_2_** reduced the severity of diarrhea by *Salmonella choleraesuis* in weaning pigs**

Item	Treatment				*P*-value
	Con	Pep	Con + Bac	Pep + Bac	
Diarrhea rate					
Day 3	0.38 ± 0.202	0.13 ± 0.125	0.13 ± 0.125	0.13 ± 0.125	0.392
Day 4	0.00 ± 0.000	0.13 ± 0.125	0.25 ± 0.164	0.25 ± 0.164	0.538
Day 5	0.14 ± 0.143^b^	0.00 ± 0.000^b^	0.62 ± 0.183^a^	0.25 ± 0.164^b^	0.026
Day 6	0.28 ± 0.184^b^	0.37 ± 0.183^b^	0.88 ± 0.125^a^	0.38 ± 0.183^b^	0.049
Day 7	0.00 ± 0.000^c^	0.38 ± 0.189^bc^	1.00 ± 0.000^a^	0.62 ± 0.183^b^	0.001
Overall	0.19 ± 0.073^b^	0.30 ± 0.065^b^	0.58 ± 0.070^a^	0.32 ± 0.100^b^	0.006

The values are the means ± SEMs (*n* = 8). The mean values within a row with different superscript letters (a–c) are significantly different (*P* < 0.05). Diarrhea index = total fecal score/total number of experimental pigs. Con: basal diet without treatment, Pep: basal diet + 50 mg/day KWH_2_ per pig, Con + Bac = basal diet + *Salmonella choleraesuis* infection, Pep + Bac: basal diet + 50 mg/day KWH_2_ per pig + *Salmonella choleraesuis* infection.

**Table 5:** **KWH**_2_** reduced the occurrence of diarrhea by *Salmonella choleraesuis* in weaning pigs**

Item	Treatment				*P*-value
	Con	Pep	Con + Bac	Pep + Bac	
Diarrhea index					
Day 3	1.21 ± 0.101	1.06 ± 0.063	1.25 ± 0.250	1.06 ± 0.063	0.702
Day 4	1.00 ± 0.000	1.06 ± 0.063	1.12 ± 0.082	1.12 ± 0.082	0.538
Day 5	1.07 ± 0.071^b^	1.00 ± 0.000^b^	1.38 ± 0.157^a^	1.16 ± 0.105^ab^	0.046
Day 6	1.14 ± 0.092^b^	1.19 ± 0.041^b^	2.19 ± 0.294^a^	1.09 ± 0.046^b^	<0.001
Day 7	1.00 ± 0.000^b^	1.38 ± 0.157^b^	2.19 ± 0.298^a^	1.50 ± 0.125^b^	0.001
Overall	1.08 ± 0.040^b^	1.14 ± 0.041^b^	1.62 ± 0.128^a^	1.16 ± 0.052^b^	<0.001

The values are the means ± SEMs (*n* = 8). The mean values within a row with different superscript letters (a–c) are significantly different (*P* < 0.05). Diarrhea index = total fecal score/total number of experimental pigs. Con: basal diet without treatment, Pep: basal diet + 50 mg/day KWH_2_ per pig, Con + Bac: basal diet + *Salmonella choleraesuis* infection, Pep + Bac: basal diet + 50 mg/day KWH_2_ per pig + *Salmonella choleraesuis* infection.

**Figure 3:**
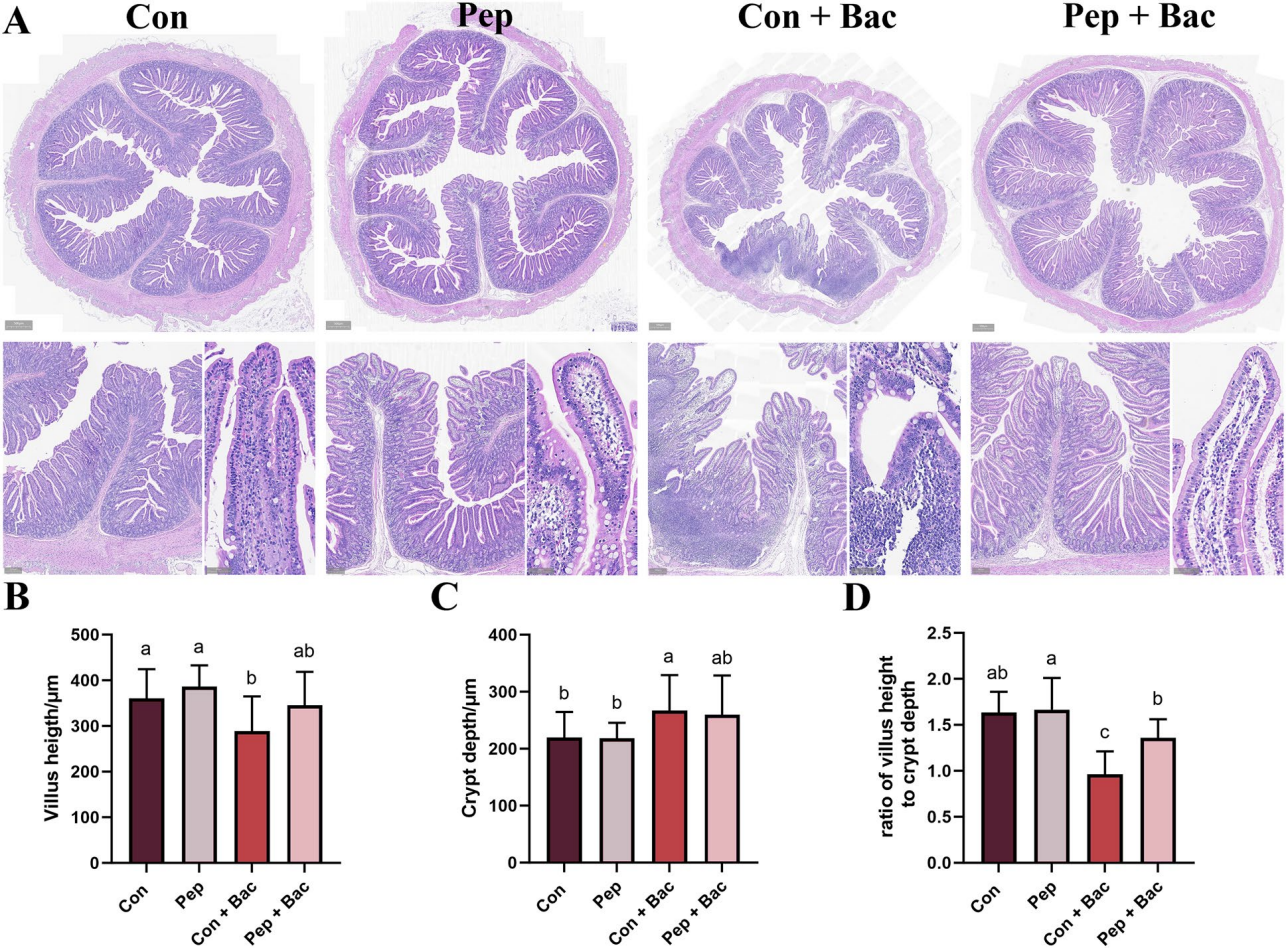
**Histological analysis of the jejunum.** **A** H&E staining of the jejunum; scale bars for the upper panel, 500 μm; scale bars for the lower left panel, 200 μm; scale bars for the lower right panel, 50 μm. **B**–**D** Villus height (**B**), crypt depth (**C**), and the ratio of villus height to crypt depth (**D**). Columns with different letters indicate significant differences (*P* < 0.05). The values are the means ± SEMs. The number of villi and crypts assessed per sample was ≥ 10. Con: basal diet without treatment, Pep: basal diet + 50 mg/day KWH_2_ per pig, Con + Bac: basal diet + *Salmonella choleraesuis* infection, Pep + Bac: basal diet + 50 mg/day KWH_2_ per pig + *Salmonella choleraesuis* infection.

The results of the histological analysis of the jejuna from the different groups are shown in Figure 3. The jejuna from the Con- and KWH_2_-treated groups (Pep and Pep + Bac) presented normal villous architecture and brush borders, with mild infiltration of inflammatory cells in the lamina propria (Figure 3A). The structure of the jejunum from the Con + Bac group was distorted, with blunt villi and expanded lamina propria. KWH_2_ protected the structure of jejunal villi from *Salmonella choleraesuis*. In addition, increased lymphocytes in the lamina propria and intraepithelium caused by *Salmonella choleraesuis* were remedied by KWH_2_. We failed to detect an effect of KWH_2_ on the villus height and crypt depth under both basal and bacterial challenge conditions (Figures 3B and C). *Salmonella choleraesuis* decreased the ratio of villus height to crypt depth (Figure 3D). KWH_2_ restored the ratio of the villus height to the crypt depth (*P* < 0.05).

**Figure 4:**
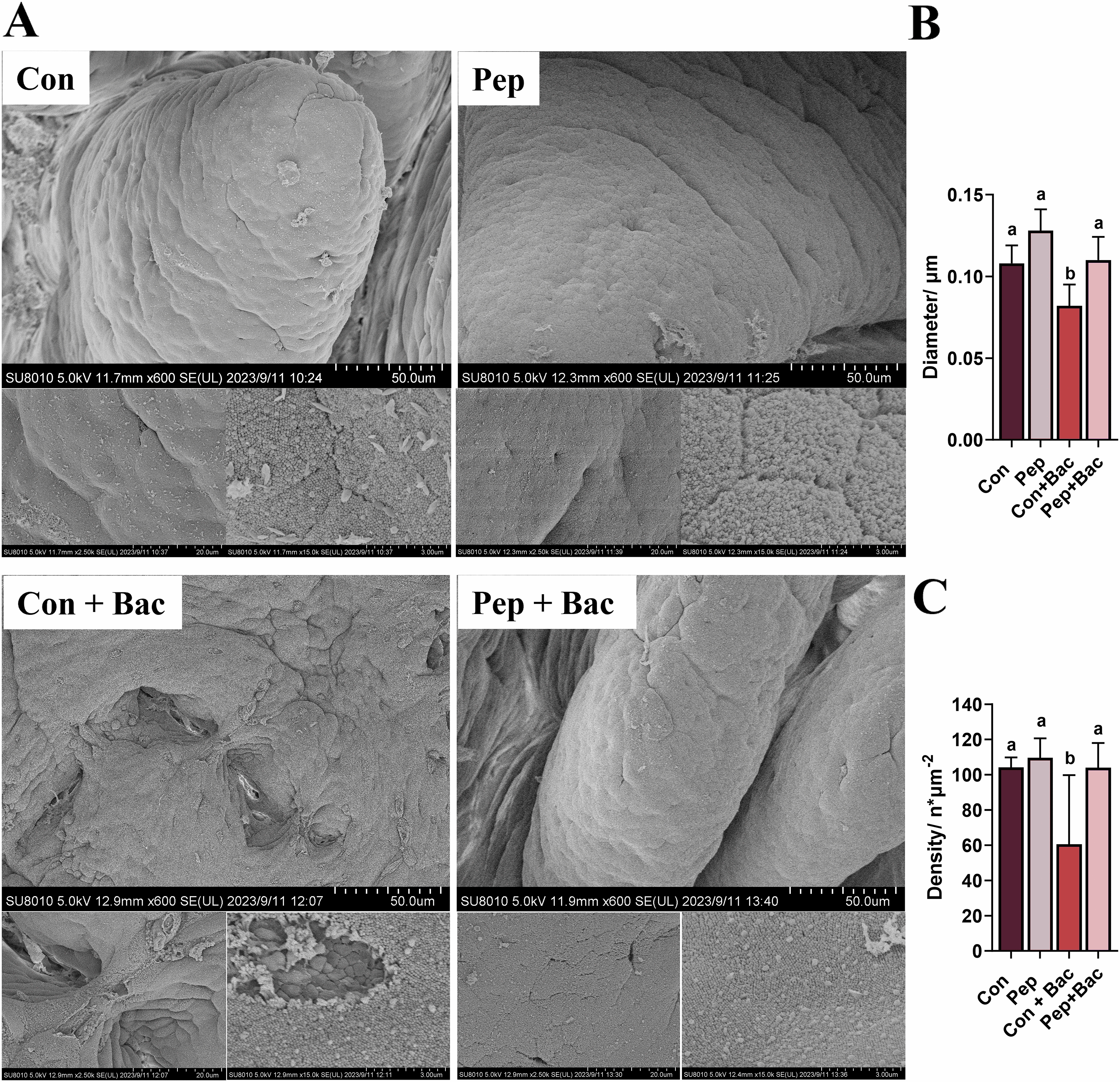
**Effects of KWH**_2_ **on the surface of jejunal villi.** **A** Surface of jejunal villi observed by scanning electron microscopy; **B** diameter of microvilli; **C** density of microvilli; (*n* ≥ 60). The values are the means ± SEMs. Columns with different letters are significantly different (*P* < 0.05). Con: basal diet without treatment, Pep: basal diet + 50 mg/day KWH_2_ per pig, Con + Bac: basal diet + *Salmonella choleraesuis* infection, Pep + Bac: basal diet + 50 mg/day KWH_2_ per pig + *Salmonella choleraesuis* infection.

The surface of the jejunal villi was evaluated by scanning electron microscopy (Figure 4). The jejunal villi were predominantly tongue-shaped in the Con, Pep, and Pep + Bac groups but were ragged in the Con + Bac group at low power (Figure 4A). Compared with those in the other groups, the microvilli in the Con + Bac group were sparser and more irregular. Under basal conditions, KWH_2_ increased the diameter of microvilli (Figure 4B; *P* < 0.05). Under challenge conditions, supplementation with KWH_2_ abolished the deleterious effects of *Salmonella choleraesuis* infection on the diameter and density of microvilli (Figure 4C; *P* < 0.05).

### Potential mechanisms by which KWH_2_ alleviates the deleterious effects of *Salmonella choleraesuis*

**Figure 5:**
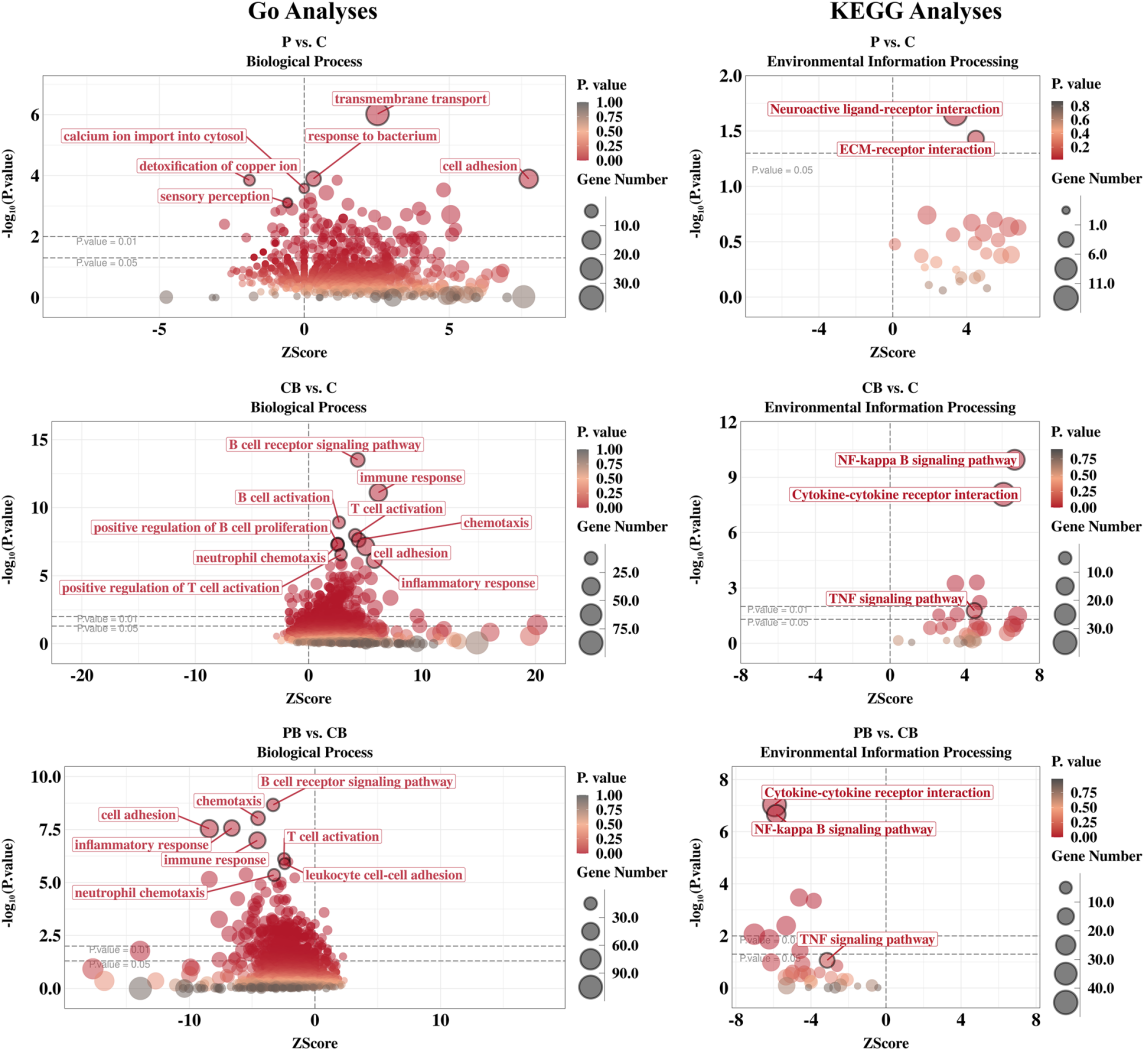
**GO and KEGG analyses of the groups.** Con: basal diet without treatment, Pep: basal diet + 50 mg/day KWH_2_ per pig, Con + Bac: basal diet + *Salmonella choleraesuis* infection, Pep + Bac: basal diet + 50 mg/day KWH_2_ per pig + *Salmonella choleraesuis* infection.

To explore the potential mechanisms of KWH_2_, we performed transcriptome analysis. Principal component analysis suggested that, compared with the other treatments, *Salmonella choleraesuis* infection resulted in greater variance in expressed genes within the group (Additional file 3). A total of 30,259 genes were detected (Additional file 4). Gene Ontology (GO) and Kyoto Encyclopedia of Genes and Genomes (KEGG) analyses were performed to assess the biological functions of KWH_2_ and the underlying mechanisms (Figure 5 and Additional file 5). Compared with the control, KWH_2_ increased the enrichment of genes related to cell adhesion, transmembrane transport, and the entry of calcium ions into the cytosol (*P* < 0.05). Bacterial challenge (Bac) increased the enrichment of genes associated with biological processes related to the response to bacteria, inflammation, and immunity (*P* < 0.05). As expected, KWH_2_ reduced the enrichment of genes related to the inflammatory response (*P* < 0.05). The results from the KEGG analysis were consistent with those from the GO analysis (Additional file 5). Under basal conditions, KWH_2_ seems to alter not only environmental information processing-related pathways but also lipid metabolism in jejunal tissue (*P* < 0.05). Bacterial challenge increased inflammation-related signaling, notably the NF-κB signaling pathway (*P* < 0.05). KWH_2_ abolished the inflammatory response induced by bacterial challenge (*P* < 0.05). On the basis of the above analysis, we demonstrated that KWH_2_ reduced the inflammatory response induced by *Salmonella choleraesuis*, but the effects of KWH_2_ seemed to differ under basal conditions (Pep) from those under bacterial challenge conditions (Pep + Bac; Additional file 5).

**Figure 6:**
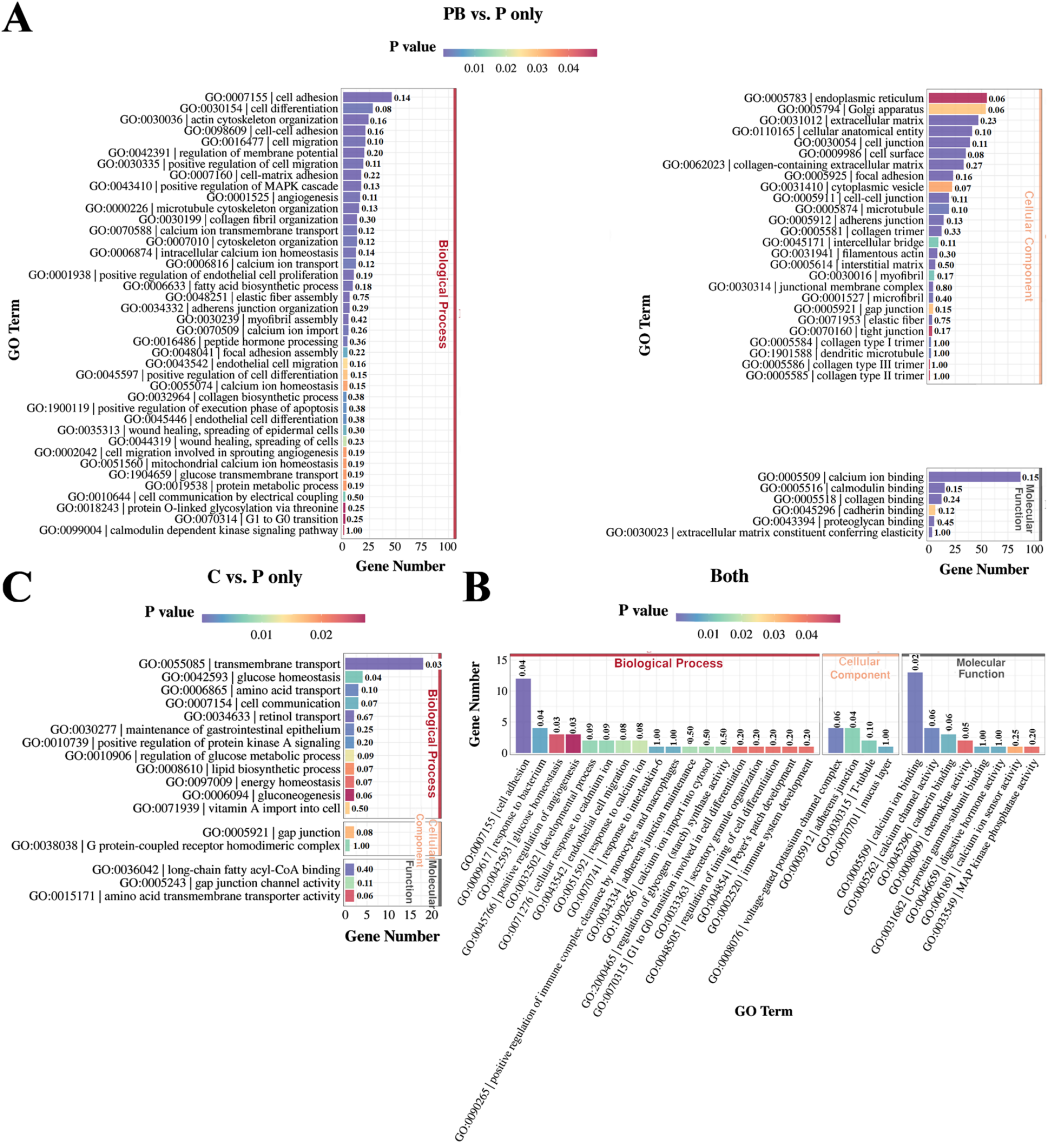
**GO analysis of the genes in the subsets of the Venn diagram (Additional file 6).** **A**–**C** GO analysis of genes differentially expressed in Pep + Bac versus Pep (**A**); Pep versus Con and Pep + Bac versus Pep (**B**); Con versus Pep (**C**). The number above the column represents the rich factor of the genes in the column. PB: Pep + Bac, C: Con, P: Pep; Con: basal diet without treatment, Pep: basal diet + 50 mg/day KWH_2_ per pig, Con + Bac: basal diet + *Salmonella choleraesuis* infection, Pep + Bac: basal diet + 50 mg/day KWH_2_ per pig + *Salmonella choleraesuis* infection.

To explore how KWH_2_ functions under basal and bacterial challenge conditions, we compared genes whose expression was altered between Pep + Bac and Pep and between Con and Pep. A total of 255 genes were altered both in Pep + Bac versus Pep and Con versus Pep; 1331 genes were differentially expressed only in Pep + Bac versus Pep; and 303 genes were differentially expressed only in Con versus Pep (Additional file 6). Gene Ontology (GO) and Kyoto Encyclopedia of Genes and Genomes (KEGG) analyses were employed on the subsets shown in Additional file 6 to explore the biological functions and pathways involved in the response to KWH_2_ under different conditions. The top enriched GO terms in both Pep + Bac versus Pep and Con versus Pep included calcium-related signaling (six terms), glucose homeostasis-related signaling (three items), and cellular process (Figure 6B). The top terms in the Con versus Pep comparison were associated mainly with glucose homeostasis (six items; Figure 6C; *P* < 0.05). The top enriched terms in the Pep + Bac versus Pep comparison included only the following categories: calcium-related signaling (ten items), cell cycle-related signaling (six items), and glucose homeostasis-related signaling (two items; Figure 6A; *P* < 0.05). On the basis of these data, we postulated that under basal conditions, KWH_2_ affects glucose homeostasis and maintenance of the gastrointestinal epithelium, whereas the effects of KWH_2_ shift from glucose homeostasis to glucose homeostasis, the cell cycle, and immunity when challenged by bacterial infection. This transition may be associated with calcium-related signaling. Considering the results from both the GO and KEGG analyses, the effects of KWH_2_ may involve two candidate pathways: the mitogen-activated protein kinase (MAPK) signaling pathway and the phosphatidylinositol 3-kinase (PI3K)–protein kinase B (Akt) signaling pathway (Additional file 6; *P* < 0.05). The MAPK and PI3K signaling pathways were selected for the following reasons: (1) both are highly conserved signal transduction networks in eukaryotic cells and intersect to orchestrate a variety of cellular processes, including metabolism and the cell cycle (proliferation, growth, and survival) [20–23], and (2) the activation of both PI3K–Akt and MAPK signaling is associated with the intracellular concentration of Ca^2+^ [24–27].

### KWH_2_ protected weaning pigs from *Salmonella choleraesuis* by phosphorylating GSK-3β to modulate glucose metabolism and downstream transcriptional signaling

**Figure 7:**
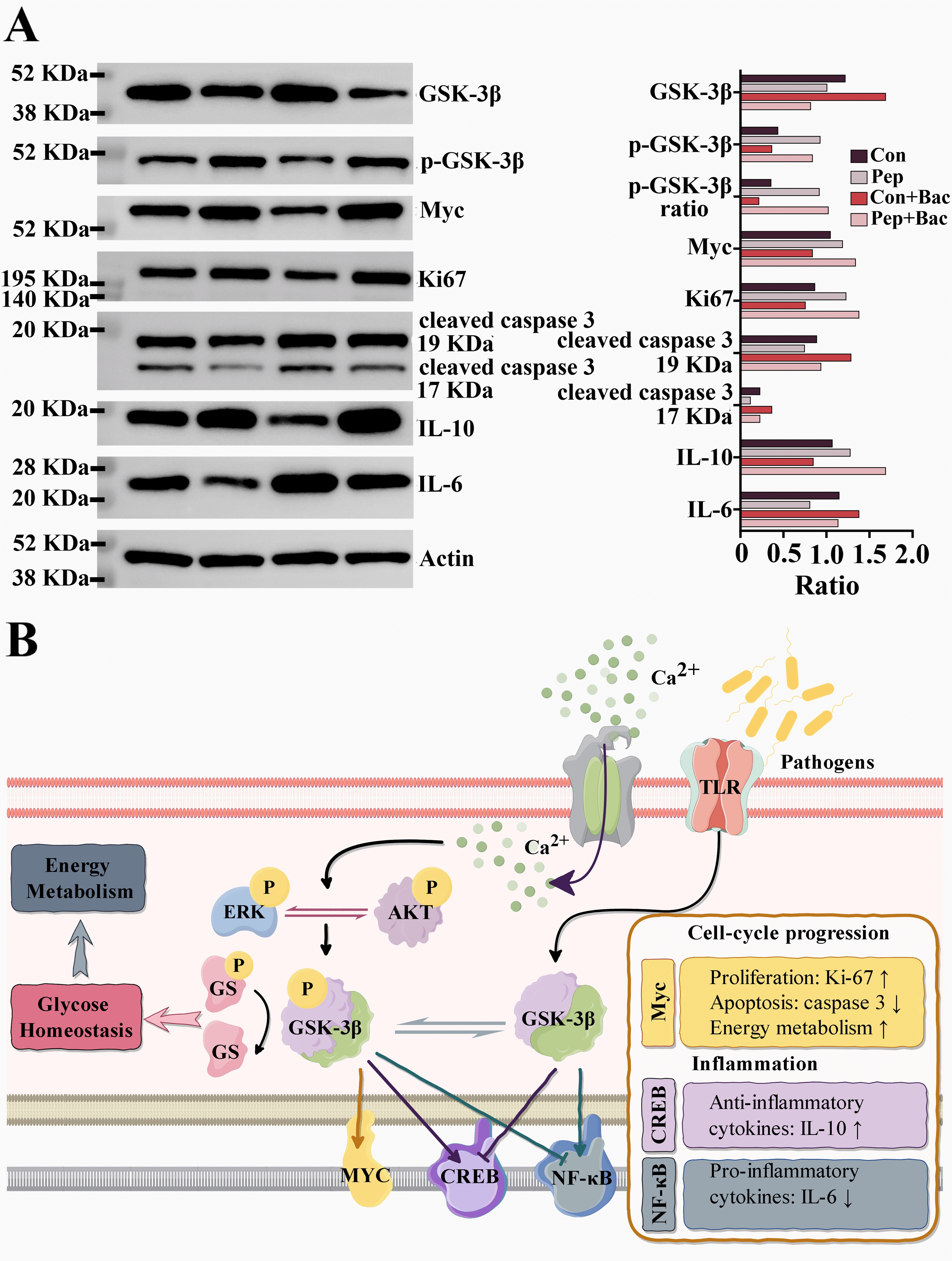
**KWH**_2_ **alleviated bacterial diarrhea by phosphorylating GSK-3β**. **A** Western blotting; each blot represents at least three independent samples. **B** Schematic diagram of how KWH_2_ functions (by Figdraw). C: Con, P: Pep, CB: Con + Bac, PB: Pep + Bac; Con: basal diet without treatment, Pep: basal diet + 50 mg/day KWH_2_ per pig, Con + Bac: basal diet + *Salmonella choleraesuis* infection, Pep + Bac: basal diet + 50 mg/day KWH_2_ per pig + *Salmonella choleraesuis* infection.

Glycogen synthase kinase 3 (GSK-3) attracted our attention according to the above analysis. GSK-3 is a kinase that lies at the convergence of the axes of MAPK and PI3K signaling. MAPK and PI3K signaling cooperate to regulate the activity of GSK-3 via phosphorylation. GSK-3 affects glucose metabolism by mechanically interacting with glycogen synthase [28]. GSK-3 has two isoforms, α and β. In addition to glycogen synthase, GSK-3β can phosphorylate transcription factors and modulate energy homeostasis, the cell cycle, and immune/inflammatory processes in response to stress, i.e., bacteria [29–31]. *NF-κB*, an effector whose expression is altered in response to KWH_2_ treatment according to transcriptome analysis, is one of the downstream transcription factors regulated by GSK-3β [32]. Western blotting suggested that (1) bacterial challenge increased the level of GSK-3β but reduced the level of p-GSK-3β, leading to a dramatic decrease in the p-GSK-3β:GSK-3β ratio (Figure 7A). KWH_2_ increased the phosphorylation of GSK-3β. As expected, the phosphorylation of GSK-3β was associated with an increase in the level of IL-10, an anti-inflammatory cytokine, and a decrease in the level of IL-6, a proinflammatory cytokine. These findings confirmed the anti-inflammatory effect of KWH_2_. Myc is a super-transcription factor family regulated by GSK-3β and is best studied for its metabolic regulation during cell cycle entry [33–35]. We noted that the level of Myc increased with the phosphorylation of GSK-3β, which was accompanied by an increase in the expression of Ki-67, a proliferation marker, and a decrease in the expression of cleaved caspase 3, an apoptosis marker. Together, we speculate that GSK-3β is an essential effector of KWH_2_. KWH_2_ regulates cell glucose metabolism, the cell cycle, and immunity/inflammation by phosphorylating GSK-3β.

Considering the above results, we postulate that KWH_2_ functions by regulating ERK and Akt signaling via calcium-related signaling (Figure 7B). Activated ERK and Akt cooperatively phosphorylate GSK-3β to modulate glucose homeostasis and downstream transcriptional signaling, including the cell cycle, immune/inflammatory processes, and energy metabolism.

## Discussion

For swine, postweaning is a period in which removing the use of antibiotics represents a challenge [36]. During the postweaning period, a major problem is the increased occurrence of diarrhea, which is most often related to bacterial infections. Various strategies have been reported to ameliorate the negative impacts of eliminating antibiotics from the diet. Among the strategies employed and investigated, prophylaxis has been preferred over treatment upon the appearance of disease problems, since symptoms of bacterial diarrhea often manifest at a late stage of infection, and the general performance of pigs is usually impaired even after recovery. Prophylactic approaches provide a chance to halt or inhibit the progression of individual infection at a meaningful early stage and avoid the onset of disease in the whole population. In this work, we investigated the potential of the bioactive peptide KWH_2_ as a prophylactic approach for weaning against diarrhea caused by *Salmonella choleraesuis*. In pigs infected with *Salmonella choleraesuis*, typical clinical manifestations of acute infection include cyanosis, dyspnea, and red-to-cyanotic discoloration of the ears, chest, and abdominal surface. Diarrhea is also a feature of *Salmonella choleraesuis* infection in piglets, a sign particularly common in immunocompromised hosts [9].

Bioactive peptides have been reported to improve the growth performance of weaning pigs [37–39]. Therefore, the effect of KWH_2_ on growth performance was investigated first. Under basal conditions, the beneficial effect of KWH_2_ on growth performance was minor and tended to increase the ADFI only from day 3 to day 7 of the 7-day treatment. One reason for this may be that (1) the dose of KWH_2_ administered was lower (50 mg/day), and/or (2) the length of trial duration (7 days) was shorter to observe beneficial effects substantially [5, 7, 40–45]. Dietary supplementation with peptide P5 (0, 40, or 60 mg/kg) or A3 (0, 60, or 90 mg/kg) had a dose-dependent beneficial effect on growth performance during the 28-day experimental period [41, 42]. Wang et al. [40] reported 41.8% and 17.20% increases in the ADG and F:G, respectively, of weaning pigs fed 1000 mg/kg lactoferrin, a milk-derived peptide, in a 15-day growth experiment. Similarly, the administration of cecropin AD to weaning pigs led to 4%, 1%, and 3% increases in ADG, ADFI, and F:G, respectively, at a dose of 400 mg/kg during a 19-day experimental period [44]. The protective effects of KWH_2_ on growth were prominent under bacterial challenge conditions, and KWH_2_ reversed the negative effects of *Salmonella choleraesuis* on growth performance. These results suggested that KWH_2_ protected weaning pigs from the negative effects of *Salmonella choleraesuis* on growth performance. We acknowledged that the clinical presentation of *Salmonella choleraesuis* is invasive, characterized by clinical manifestations of enterocolitis and septicemia. The early signs of septicemia include hyperthermia, inappetence, lethargy, and increased respiratory rate; while late-stage signs involve dyspnea, cyanosis, hypothermia, and even sudden death. During the experiment, we did observe that pigs infected with *Salmonella choleraesuis* showed inappetence and a tendency toward lethargy. Inappetence was evidenced by reduced feed intake. Lethargy is a more subjective behavioral parameter, commonly assessed by recording behavior over a set period and quantifying it. Since the focus of our study was on intestinal barrier function, its morphology, and underlying molecular mechanisms, we did not evaluate lethargy. No morbidity was detected in infected pigs. One reason for this is that the morbidity rate associated with *Salmonella choleraesuis* is time dependent. During acute outbreak of postweaning diarrhea, mortality among infected pigs may reach 20–30% over a 1–2-month time frame [9]. Given the shorter duration of our experiment, it is reasonable that no mortality was observed.

The effects of *Salmonella choleraesuis* on the occurrence and severity of diarrhea started to be overt from day 5, the second day of a 4-day infection. This aligns with the pathogenesis of *Salmonella choleraesuis* infection. As the bacteria invade the intestinal epithelium, they trigger a local inflammatory response. The invasion of bacteria and the infiltration of immune cells during inflammation leads to direct damage of intestinal integrity, disruption of normal functions of intestine, impairment of water absorption, and onset of diarrhea. Septicemia arises from a severe systemic inflammatory response, which occurs at a later stage than local inflammation. This observation suggests that the model in the study was at the stage of initial systemic dissemination. On the basis of the photos taken, as the consistency of feces decreased, the color of the feces became yellowish, a sign of *Salmonella choleraesuis* infection [46]. In agreement with the results from growth performance, KWH_2_ effectively abated the detrimental effects of *Salmonella choleraesuis*, suggesting the efficiency of KWH_2_ in preventing bacterial diarrhea in weaning pigs. The diarrhea data revealed an inconsistent effect of KWH_2_ on the occurrence and severity of diarrhea on days 5 and 7. Day 5 was the first day on which the effects of *Salmonella choleraesuis* on diarrhea appeared, and day 7 was the last day of the trial. Weaning diarrhea is a complex phenomenon with multiple factors, including nutritional, physiological, and environmental contributing factors [47]. Typically, approximately 10% of the population may suffer from slight diarrhea for various reasons, e.g., separation from the sow, mixing litters, and switching from milk to feed. In the early phase of infection, the effects of *Salmonella choleraesuis* on the severity of diarrhea were moderate; the influence of other contributing factors was apparent; thus, the results were affected. The comparable occurrence of diarrhea among the Pep + Bac, Pep, and Con groups demonstrated the effectiveness of KWH_2_. However, at the later phase of infection, pigs suffer from severe and persistent diarrhea. The severity of diarrhea is a more persuasive parameter for assessing the effectiveness of KWH_2_. Taken together, these findings suggest that KWH_2_ effectively protects pigs from *Salmonella choleraesuis*-induced diarrhea.

Diarrhea is associated with an increase in fluids to the intestinal lumen due to increased epithelial permeability. An increase in epithelial permeability is a sign of destruction of the intestinal epithelial barrier. Plasma DAO and d-lactic acid are two indices that reflect the permeability of the intestinal wall [48]. DAO is an intracellular enzyme present in intestinal villous cells. When the integrity of the intestinal epithelium is disrupted, intracellular DAO is released into the circulation. d-Lactic acid is a metabolic end product of intestinal bacteria. Upon destruction of the intestinal barrier, d-lactic acid infiltrates portal blood. Therefore, DAO and d-lactic acid are normally employed as indicators of increased permeability of the intestinal epithelium. The administration of KWH_2_ abolished the effect of *Salmonella choleraesuis* on the serum DAO and d-lactic acid levels, suggesting the efficiency of KWH_2_ in reducing the vulnerability of the intestinal epithelium to bacterial infection.

The jejunum is an important site for the digestion and absorption of nutrients. The jejunum accounts for nearly 80% of the small intestine in pigs, where feeds are mostly digested to nutrients and then absorbed [49]. Under stress, the intestinal morphology changes. KWH_2_ had no effect on the villus height, crypt depth, or the villus/crypt ratio under basal conditions. *Salmonella choleraesuis* resulted in distorted villous architecture, with reduced villus height, increased crypt depth, and a decreased ratio of villus height:crypt depth, all suggesting a destructive effect of *Salmonella choleraesuis* on intestinal morphology. KWH_2_ did not affect villus height or crypt depth but alleviated the reduction in the ratio of villus height:crypt depth under bacterial challenge conditions. An increased ratio of villus height:crypt depth reflects an increase in epithelial turnover and greater intestinal absorption of nutrients, suggesting that KWH_2_ combats bacterial infection by promoting nutrient absorptive capacity and the beneficial effect of KWH_2_ on intestinal health [43, 44, 50]. By using bioactive peptides, Tang [7] and colleagues reported an increase in the villus height of the jejunum, which we were unable to detect. A reason for this may be that, in our study, the villus heights were measured from intact villi. Many villi in the Con + Bac group were desquamated and not counted. Hence, the beneficial effect of KWH_2_ might be overshadowed under bacterial challenge conditions. *Salmonella choleraesuis* resulted in an obvious inflammatory response in the jejunum. KWH_2_ reduces the amplitude of inflammation in the jejunum, highlighting its anti-inflammatory role [37, 41, 42, 51]. As expected, scanning electron microscopy confirmed that KWH_2_ promoted resistance to bacterial damage to the intestinal epithelium.

To investigate the underlying mechanism of KWH_2_, a transcriptome analysis was performed. Principal component analysis revealed greater variation in the *Salmonella choleraesuis* group. This was due to individual differences in stress susceptibility and stress response mechanisms [52]. GO analysis was performed to understand the biological functions regulated by KWH_2_. Under basal conditions, KWH_2_ was enriched in genes that regulate transmembrane transport, cell adhesion, and ion channel activity. Consistent with previously reported studies [53], *Salmonella choleraesuis* infection increased the number of genes enriched in immune/inflammatory responses and lipopolysaccharide-mediated signaling pathways. KWH_2_ reduced the inflammatory response to *Salmonella choleraesuis* infection, suggesting an anti-inflammatory role. The genes that were differentially expressed between Pep + Bac and Con were enriched in lipid metabolism. KEGG analysis revealed that *Salmonella choleraesuis* elevated the NF-κB signaling pathway, but the mechanism of KWH_2_ was quite obscure and seemed to be correlated mainly with metabolism. To clarify the mechanism of KWH_2_, we compared genes altered in Con versus Pep and Pep + Bac versus Pep. GO analysis suggested a transition from glucose homeostasis to cell physiological activity (e.g., the cell cycle and metabolism) when *Salmonella choleraesuis* with calcium-related signaling was used throughout the process. We postulated that KWH_2_ functions by regulating cell metabolism and biology in response to bacterial stimuli via calcium-related signaling. KWH_2_ contains a motif that can interact with G protein-coupled receptors, a group of membrane proteins that control calcium dynamics and are present in most cell types [54, 55]. We previously reported that KWH_2_ altered the level of intracellular calcium in vivo [8]. During bacterial infection, cells normally undergo apoptosis or exhaustion. A delicate balance between proliferation and apoptosis is needed for cells to cope with inflammatory stress and survive [3]. High metabolic activity is required for biosynthesis to initiate the cell cycle and to support the function and morphology of the intestinal epithelium. Facing stress, decreased energy intake in piglets induced a reduction in energy metabolism and dysfunction in jejunal epithelial cells [38]. The arrest of the cell cycle and the inhibition of cell proliferation in the jejunum aggravated weaning diarrhea [56, 57]. In addition, metabolism steers and coordinates the generation of wound-associated epithelial cells, a cell type required for efficient barrier restoration upon injury [58]. Metabolic flexibility is critical to the renewal and regeneration capacity of the intestinal epithelium. Hence, we suggest that KWH_2_ may increase resistance to inflammatory triggers by modulating metabolism. Glucose is an important source of energy for epithelial cells in the intestine. Therefore, one potential mechanism may be that KWH_2_ regulates glucose homeostasis to modulate cell metabolism, enhance intestinal integrity, and reduce vulnerability to bacterial infection. In addition, the first defense barrier between the host and pathogenic bacteria is composed of intestinal epithelial cells and mucous layers. The mucus is composed predominantly of mucin glycoproteins. Reduced secretion of mucin is one of the reasons for weaning diarrhea in piglets [59]. Mucins are a diverse family of densely glycosylated proteins that aid rapid bacterial clearance [60]. Glycosylation is a posttranscriptional process dynamically regulated by metabolic flux, e.g., glucose [61]. The genes that were altered in both the Pep + Bac versus Pep and Con versus Pep groups were enriched in the mucus layer (in the cellular component category), together with glucose homeostasis-related biological processes. H&E staining revealed that the density of goblet cells, a cell type that secretes mucins, was greater in the KWH_2_-treated samples than in their corresponding counterparts (Pep versus Con and Pep + Bac versus Con + Bac). An increase in intestinal permeability reflects destruction of the intestinal mucosal barrier [62–64]. We suggest that KWH_2_ promotes the repair of the damaged intestinal barrier by (1) protecting the integrity of intestinal epithelial cells and (2) increasing the secretion of mucin, possibly by regulating glucose homeostasis.

According to the KEGG analysis, two signaling pathways, MAPK and PI3K, were identified. These two signals can (1) be regulated by Ca^2+^ and (2) transduce Ca^2+^ signals into responses encompassing the majority of outputs from GO analysis, e.g., the cell cycle (proliferation, growth, and survival), metabolism, and immunity [20–23]. These two signals are involved in adapting the response mechanism to bacterial attacks and seem to be the chief mechanism of KWH_2_. GSK-3β is a common downstream component of PI3K–Akt and MAPK signaling and is known to regulate glucose metabolism, protein synthesis, and gene transcription to modulate cellular metabolism, the cell cycle, and immunity/inflammation [29–31, 65]. GSK-3 influences physiology and pathology by phosphorylating its substrates. Activation of Akt and ERK (classic MAPKs) has been reported to phosphorylate GSK-3β and reduce the activity of GSK-3β [66, 67]. Upon bacterial infection, a timely rise of IL-6 is necessary for eliminating the pathogen, but persistent IL-6 signaling disrupts tight junctions and damages intestinal integrity. IL-10 counteracts inflammation by suppressing immune cell activation, enhancing barrier function, and promoting epithelial cell survival. Ideally, an early IL-6 peak should be followed by a timely IL-10-dominated resolution phase, ensuring controlled inflammation without collateral damage. Western blot results suggested that inhibition of GSK-3β was associated with reduced IL-6 expression and increased IL-10 expression at the end of the experiment, suggesting that the inhibition of GSK-3β reduces inflammation and improves resistance to injury caused by bacterial infection [66, 68]. Long-term activation of GSK-3β results in deleterious inflammation and reduced regenerative capacity [69]. Myc is a super-transcription factor family regulated by GSK-3β, and the protein expression of Myc is tightly correlated with metabolism and cell proliferation rates [70]. Depletion of Myc inhibited proliferation and arrested the cell cycle [71]. The western blotting results suggested a role for GSK-3β phosphorylation in the effects of KWH_2_ on cell glucose metabolism, the cell cycle, and immunity.

One point to note is that the findings of this study are preliminary regarding industrial application. The long-term effectiveness and dose–response relationship of KWH_2_ require further evaluation. Future research should focus on investigating KWH_2_’s effects over longer observation periods and across multiple dose levels to solidify KWH_2_’s potential for field application.

## Conclusions

This study reported a multifunctional bioactive peptide, KWH_2_, that can be employed as a prophylactic agent to prevent bacterial diarrhea. KWH_2_ protected growth performance, intestinal permeability, and intestinal morphology from the deleterious effects of pathogenic bacteria. KWH_2_ modulated glucose homeostasis, the cell cycle, and immunity in intestinal epithelial cells. The underlying mechanism involves the phosphorylation of GSK-3β. Phosphorylation of GSK-3β was accompanied by reduced inflammation, an accelerated cell cycle, and increased expression of Myc. This study demonstrated the efficiency and safety of KWH_2_ in preventing bacterial diarrhea in weaning pigs, providing a drug candidate for preventing/treating infectious diarrhea. Revealing the molecular mechanisms involved in the defense against/recovery from bacterial diarrhea paves the way for the design of drugs that can combat bacterial infection and enhance intestinal integrity in medical applications.

## Supplementary Information

**Additional file 1. Chemical structural formula and matrix-assisted laser desorption/ionization time-of-flight mass spectrometryspectra of the KWH**_**2**_**.**

**Additional file 2. High-performance liquid chromatographyspectra of the KWH**_**2**_. HPLC for deterring the purity and the stability of synthesized KWH_2_. For testing the stability of KWH_2_, 1 mg/mL KWH_2_ was mixed with same volume of gastric juice/intestinal juice, incubated for different time intervals, and then underwent HPLC analysis.

**Additional file 3. Principal components analysis of genes expressed in jejunal tissue among groups.**

**Additional file 4. Differentially expressed genes among groups.**summary of differentially expressed genes in different comparisons.Volcano plots representing differentially expressed genes.

**Additional file 5. Go and KEGG enrichment of differentially expressed genes between treatments.**

**Additional file 6. Venn diagram of differently expressed genes in Pep + Bac versus Pep and Pep versus Con, and KEGG analysis based on the genes in subsets of Venn diagram**. For KEGG enrichment, number above column showing the rich factor of the genes in the column.

**Additional file 7. Go analysis for genes differently expressed in Pep + Bac versus Pep Only, both in Pep + Bac versus Pep and Con versus Pep, and Con versus Pep only**. S: Significant ID number; TS: Total Significant ID Number; B: Background ID Number; ID Number; Rich Factor =S/B. Sig. Sign. *P* = Significant sign based on *P* value.

## Data Availability

The data analyzed during the current study are available from the corresponding author upon reasonable request.

## Abbreviations

RP-HPLC: reversed-phase high-performance liquid chromatography
MALDI-TOF MS: matrix-assessed laser desorption/ionization time-of-flight mass spectrometry
SEM: standard error of the mean
MIC: minimum inhibitory concentration
ADG: average daily gain
ADFI: average daily feed intake
F:G: feed-to-gain ratio
d-Lac: d-Lactic acid
DAO: diamine oxidase
H&E: hematoxylin and eosin
SDS‒PAGE: sodium dodecyl sulfate‒polyacrylamide gel electrophoresis
GSK-3: glycogen synthase kinase 3
p-GSK-3: phosphorylated glycogen synthase kinase 3
IL-6: interleukin-6
IL-10: interleukin-10
Myc: *Myc* proto-oncogene
Ki-67: *MKI67* gene
PI3K: phosphoinositide 3-kinase
MAPK: mitogen-activated protein kinase
