# Supplementary material for: Dual-action peptide KWH2 protects against Salmonella choleraesuis diarrhea in weaned piglets by enhancing intestinal barrier integrity and modulating GSK-3β/Myc signaling
Source: Vet Res. 2026 Mar 17;57:53. doi: 10.1186/s13567-025-01682-x (PMC13104273; doi:10.1186/s13567-025-01682-x)
Supplement: Supplementary file 1 — Additional file 1. Chemical structural formula and matrix-assisted laser desorption/ionization time-of-flight mass spectrometryspectra of the KWH2. [file 13567_2025_1682_MOESM1_ESM.docx]

**Additional file 1 Chemical structural formula and matrix-assisted laser desorption/ionization time-of-flight mass spectrometry (MALDI-TOF MS) spectra of the KWH_2._**


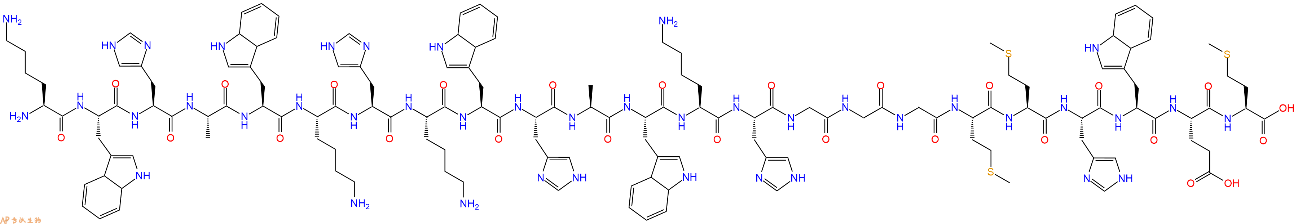


**Chemical structural formula and sequence of the KWH_2_**


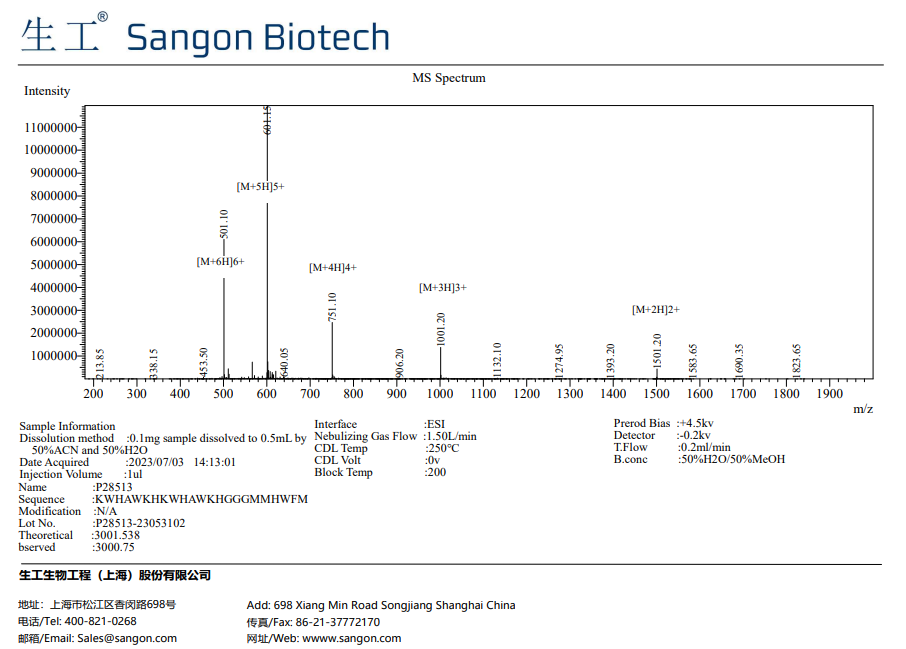


**Matrix-assisted laser desorption/ionization time-of-flight mass spectrometry (MALDI-TOF MS) spectra of the KWH_2_.**
