# Supplementary material for: Dual-action peptide KWH2 protects against Salmonella choleraesuis diarrhea in weaned piglets by enhancing intestinal barrier integrity and modulating GSK-3β/Myc signaling
Source: Vet Res. 2026 Mar 17;57:53. doi: 10.1186/s13567-025-01682-x (PMC13104273; doi:10.1186/s13567-025-01682-x)
Supplement: Supplementary file 2 — Additional file 2. High-performance liquid chromatographyspectra of the KWH2. HPLC for deterring the purity and the stability of synthesized KWH2. For testing the stability of KWH2, 1 mg/mL KWH2 was mixed with same volume of gastric juice/intestinal juice, incubated for different time intervals, and then underwent HPLC analysis. [file 13567_2025_1682_MOESM2_ESM.docx]

**Additional file 2 High-performance liquid chromatography (HPLC) spectra of the KWH_2_.**

**
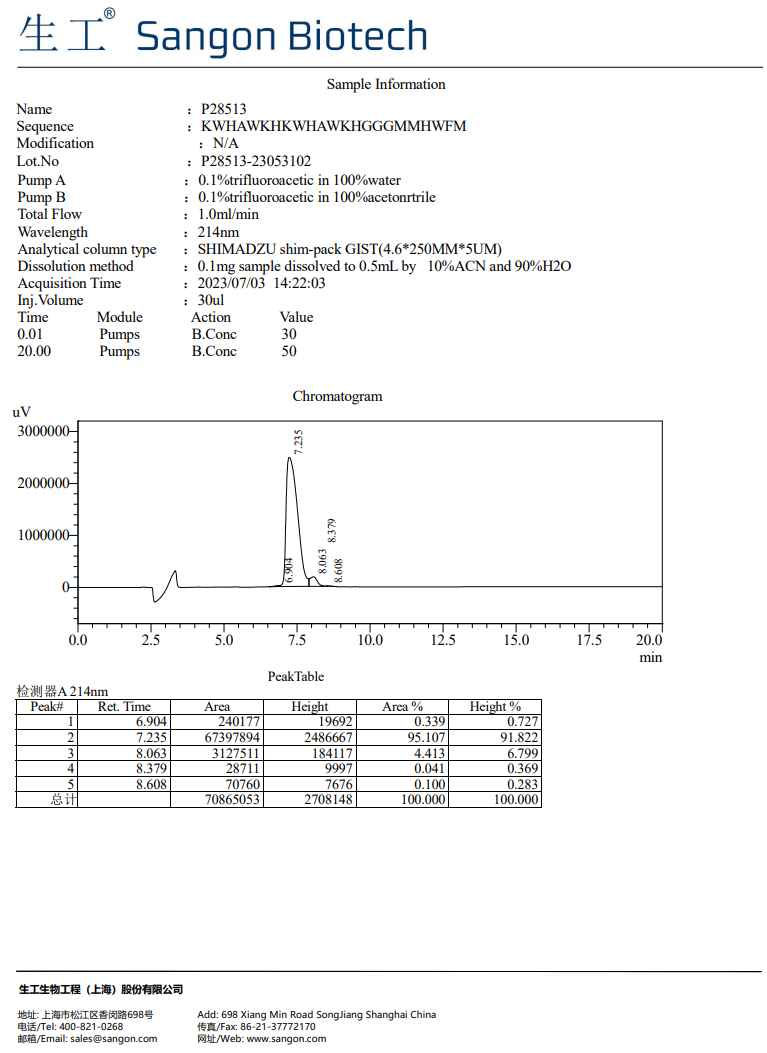
**

**HPLC for deterring the purity of synthesized KWH_2_**.


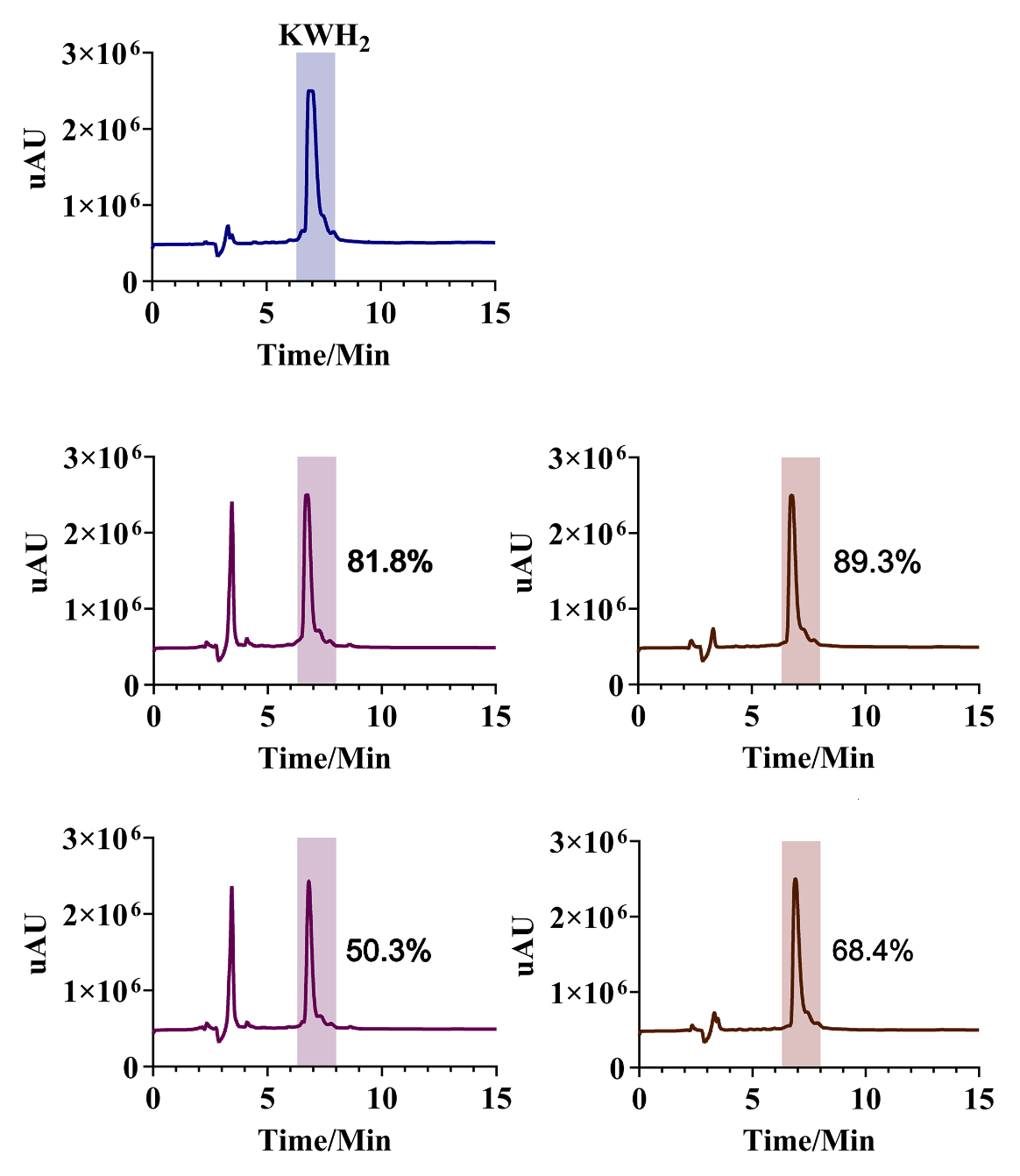


**Gastric Juice**

**Intestinal Juice**

**o.5 h**

**1 h**

**HPLC for the stability of KWH_2_.** 1 mg/mL KWH_2_ was mixed with same volume of gastric juice/ intestinal juice, incubated for different time intervals, and then underwent HPLC analysis
