# Supplementary material for: Dual-action peptide KWH2 protects against Salmonella choleraesuis diarrhea in weaned piglets by enhancing intestinal barrier integrity and modulating GSK-3β/Myc signaling
Source: Vet Res. 2026 Mar 17;57:53. doi: 10.1186/s13567-025-01682-x (PMC13104273; doi:10.1186/s13567-025-01682-x)
Supplement: Supplementary file 3 — Additional file 3. Principal components analysis of genes expressed in jejunal tissue among groups. [file 13567_2025_1682_MOESM3_ESM.docx]

**Additional file 3 Principal Components Analysis of genes expressed in jejunal tissue among groups**


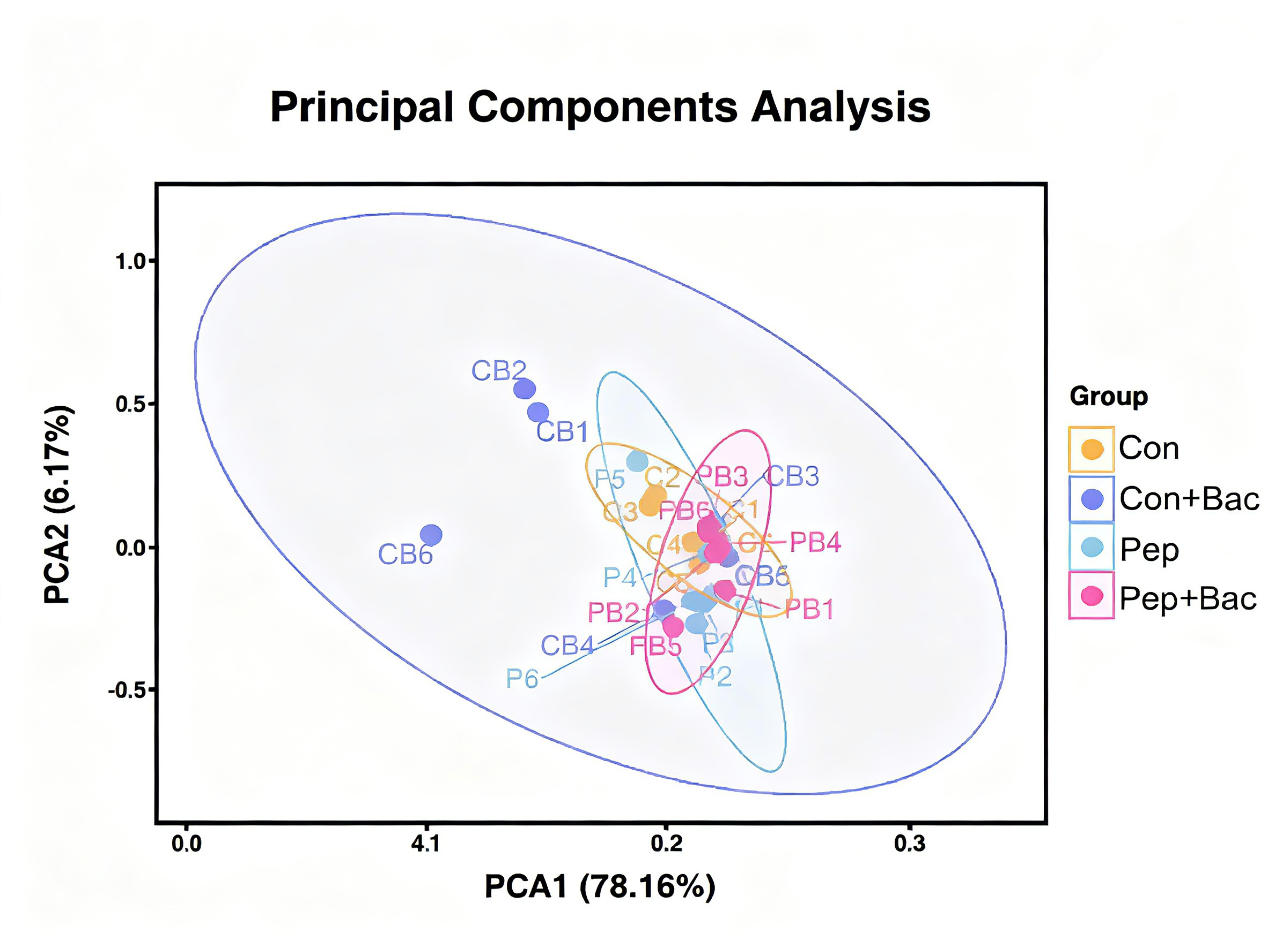


**Principal Components Analysis of genes expressed in jejunal tissue among groups.** C: Control; CB: Con+Bac; P: Pep; PB: Pep + Bac
