# Supplementary material for: Dual-action peptide KWH2 protects against Salmonella choleraesuis diarrhea in weaned piglets by enhancing intestinal barrier integrity and modulating GSK-3β/Myc signaling
Source: Vet Res. 2026 Mar 17;57:53. doi: 10.1186/s13567-025-01682-x (PMC13104273; doi:10.1186/s13567-025-01682-x)
Supplement: Supplementary file 4 — Additional file 4. Differentially expressed genes among groups.summary of differentially expressed genes in different comparisons.Volcano plots representing differentially expressed genes. [file 13567_2025_1682_MOESM4_ESM.docx]

**Additional file 4 Differentially expressed genes among groups.**


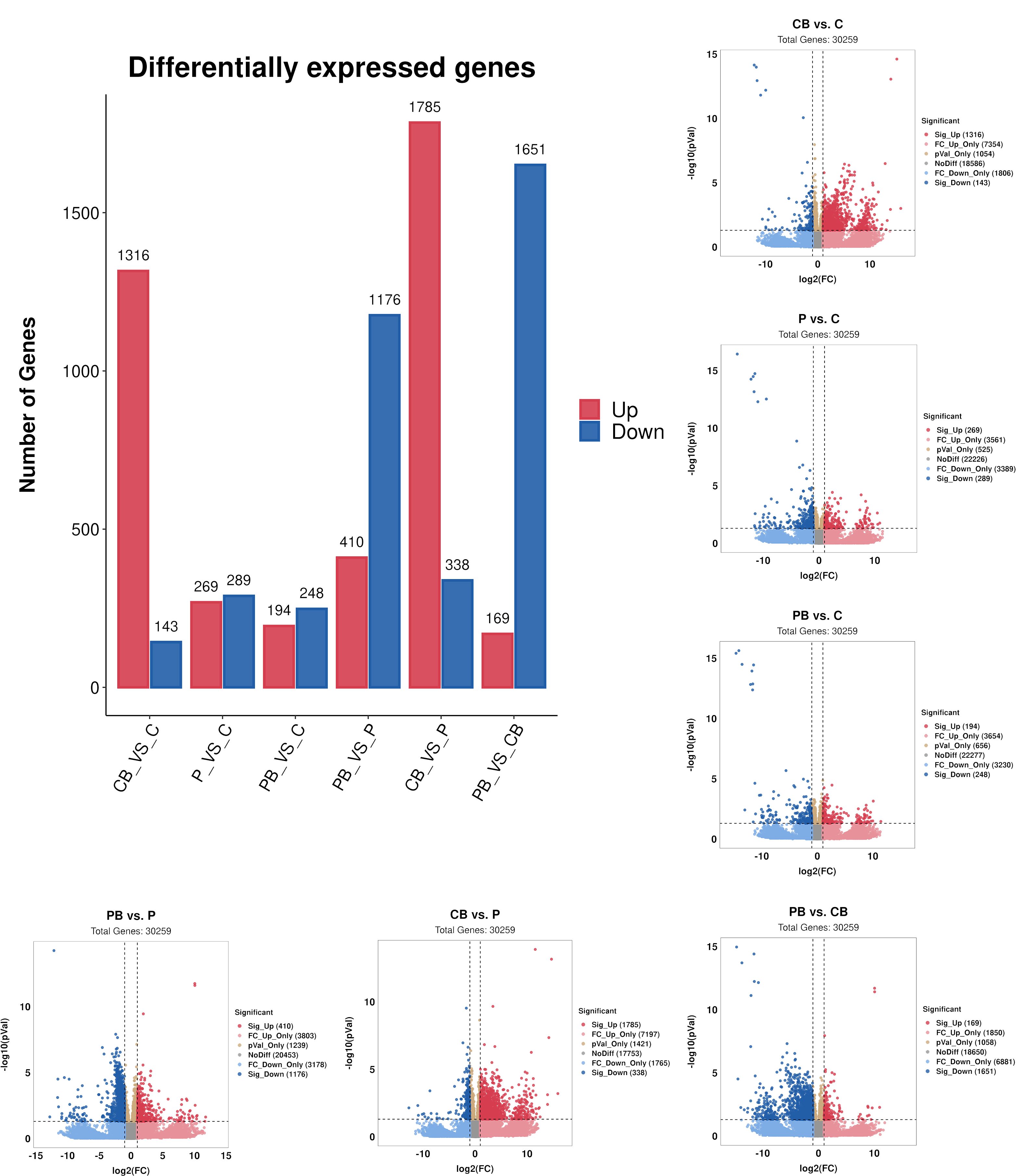


**(B)**

**(C)**

**(D)**

**(G)**

**(F)**

**(E)**

**(A)**

**Differentially expressed genes among groups.** (A) summary of differentially expressed genes in different comparisons. (B-G) Volcano plots representing differentially expressed genes of Con + Bac vs. Con (B); Pep vs. Con (C); Pep + Bac vs. Con (D); Pep + Bac vs. Pep (E); Con + Bac vs. Pep (F); Pep + Bac vs. Con + Bac (G). CB: Con + Bac, PB: Pep + Bac, CB: Con + Bac, PB: Pep + Bac
