# Supplementary material for: Dual-action peptide KWH2 protects against Salmonella choleraesuis diarrhea in weaned piglets by enhancing intestinal barrier integrity and modulating GSK-3β/Myc signaling
Source: Vet Res. 2026 Mar 17;57:53. doi: 10.1186/s13567-025-01682-x (PMC13104273; doi:10.1186/s13567-025-01682-x)
Supplement: Supplementary file 5 — Additional file 5. Go and KEGG enrichment of differentially expressed genes between treatments. [file 13567_2025_1682_MOESM5_ESM.docx]

**Additional file 5 Go and KEGG enrichment of differentially expressed genes between treatments**


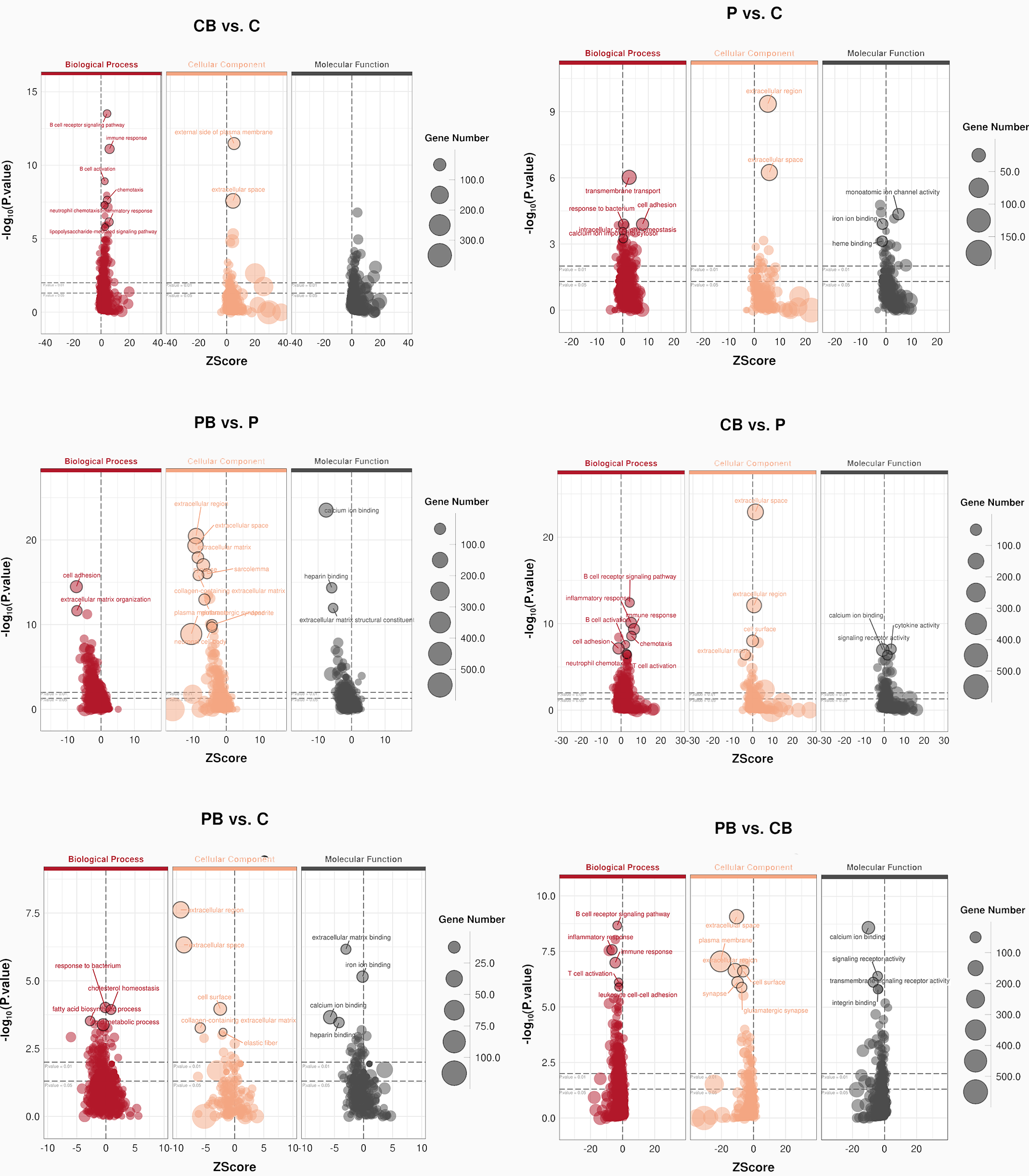


**Go enrichment of differentially expressed genes between treatments.** CB: Con + Bac, PB: Pep + Bac, CB: Con + Bac, PB: Pep + Bac


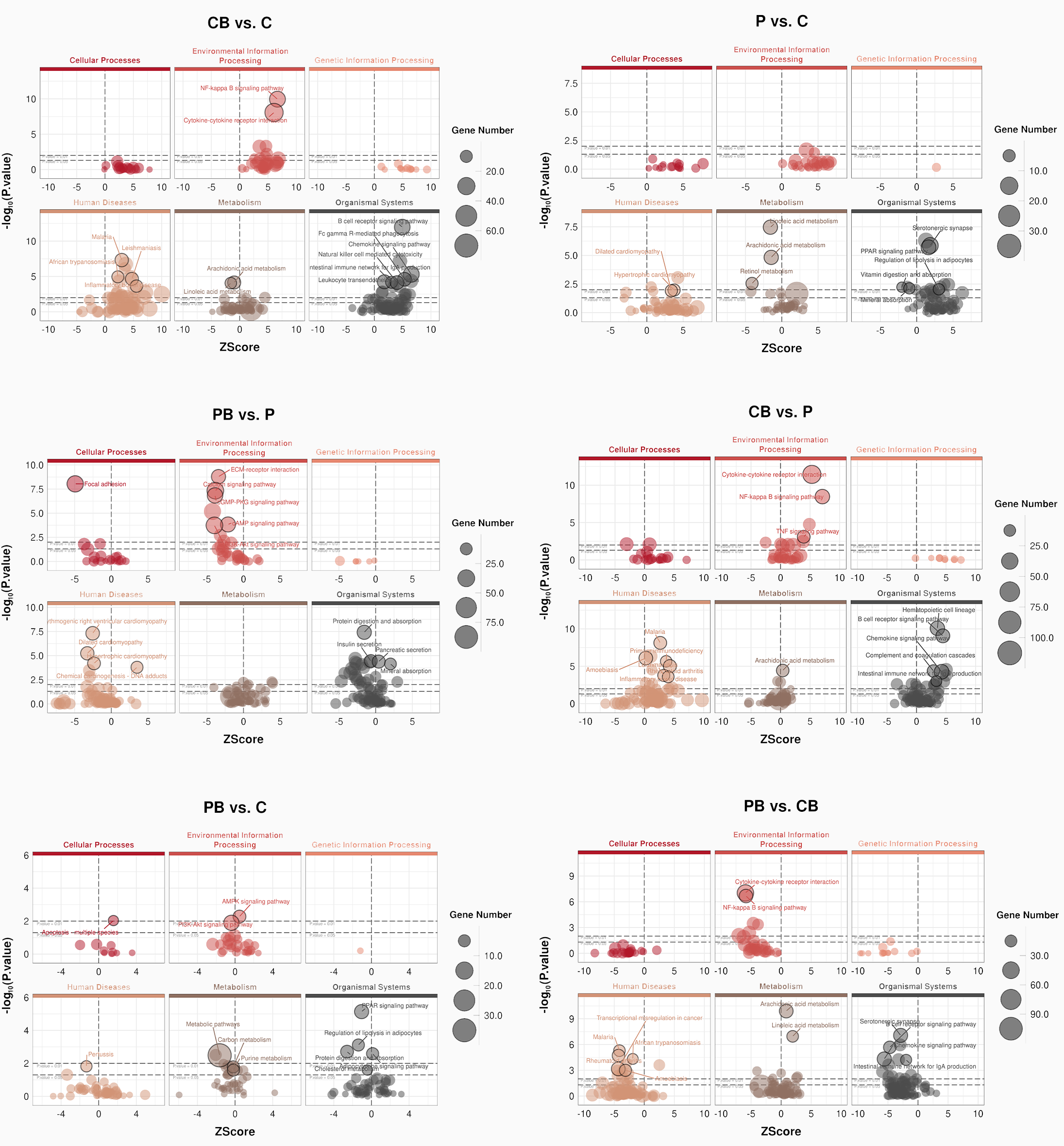


**KEGG enrichment of differentially expressed genes between treatments.** CB: Con + Bac, PB: Pep + Bac, CB: Con + Bac, PB: Pep + Bac
