# Supplementary material for: Dual-action peptide KWH2 protects against Salmonella choleraesuis diarrhea in weaned piglets by enhancing intestinal barrier integrity and modulating GSK-3β/Myc signaling
Source: Vet Res. 2026 Mar 17;57:53. doi: 10.1186/s13567-025-01682-x (PMC13104273; doi:10.1186/s13567-025-01682-x)
Supplement: Supplementary file 6 — Additional file 6. Venn diagram of differently expressed genes in Pep + Bac versus Pep and Pep versus Con, and KEGG analysis based on the genes in subsets of Venn diagram. For KEGG enrichment, number above column showing the rich factor of the genes in the column. [file 13567_2025_1682_MOESM6_ESM.docx]

**Additional file 6 Venn diagram of differently expressed genes in Pep + Bac vs. Pep and Pep vs. Con, and KEGG analysis based on the genes in subsets of Venn diagram.**


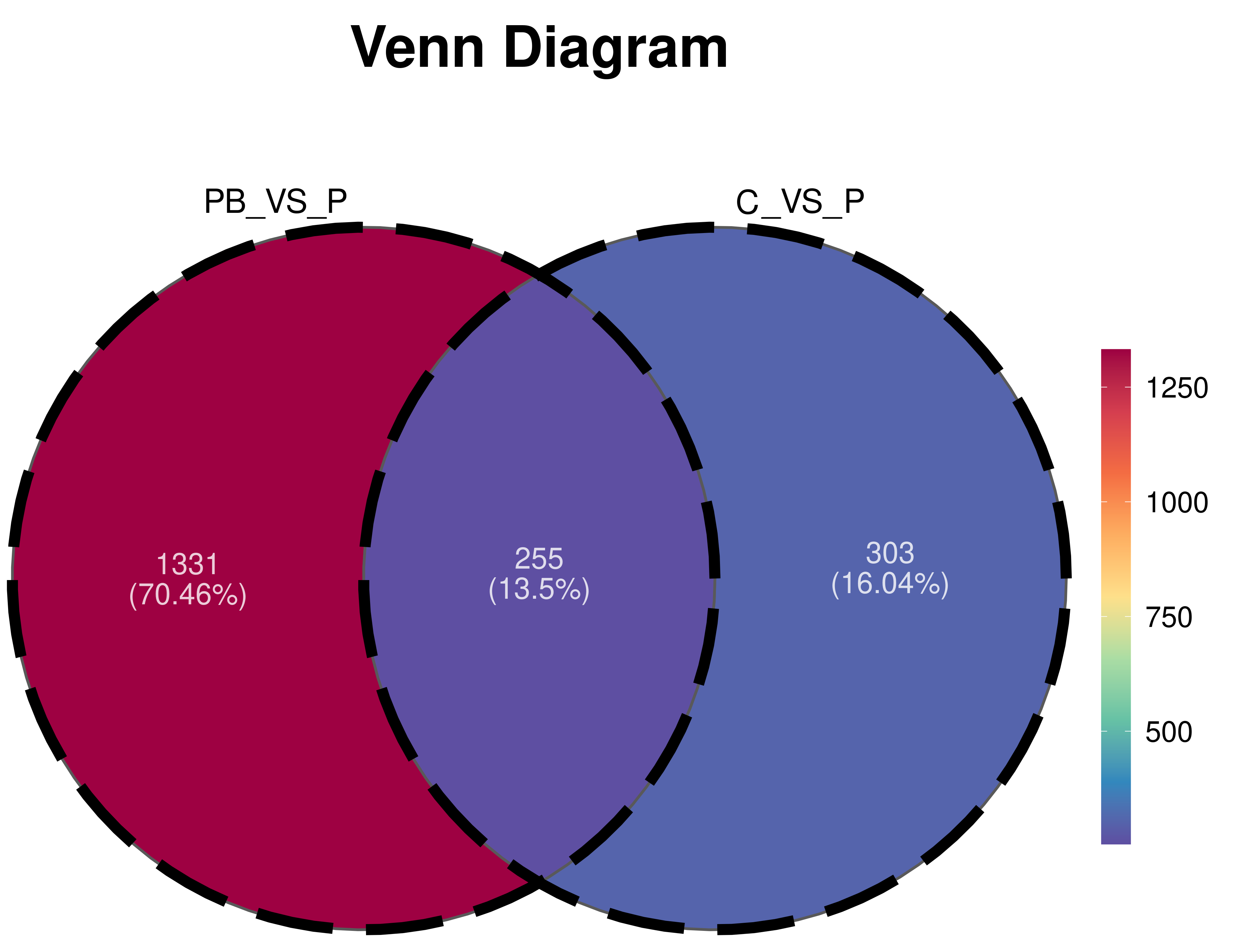


**Venn diagram** **of differently expressed genes** in Pep + Bac vs. Pep and Pep vs. Con. PB: Pep + Bac; C: Con; P: Pep


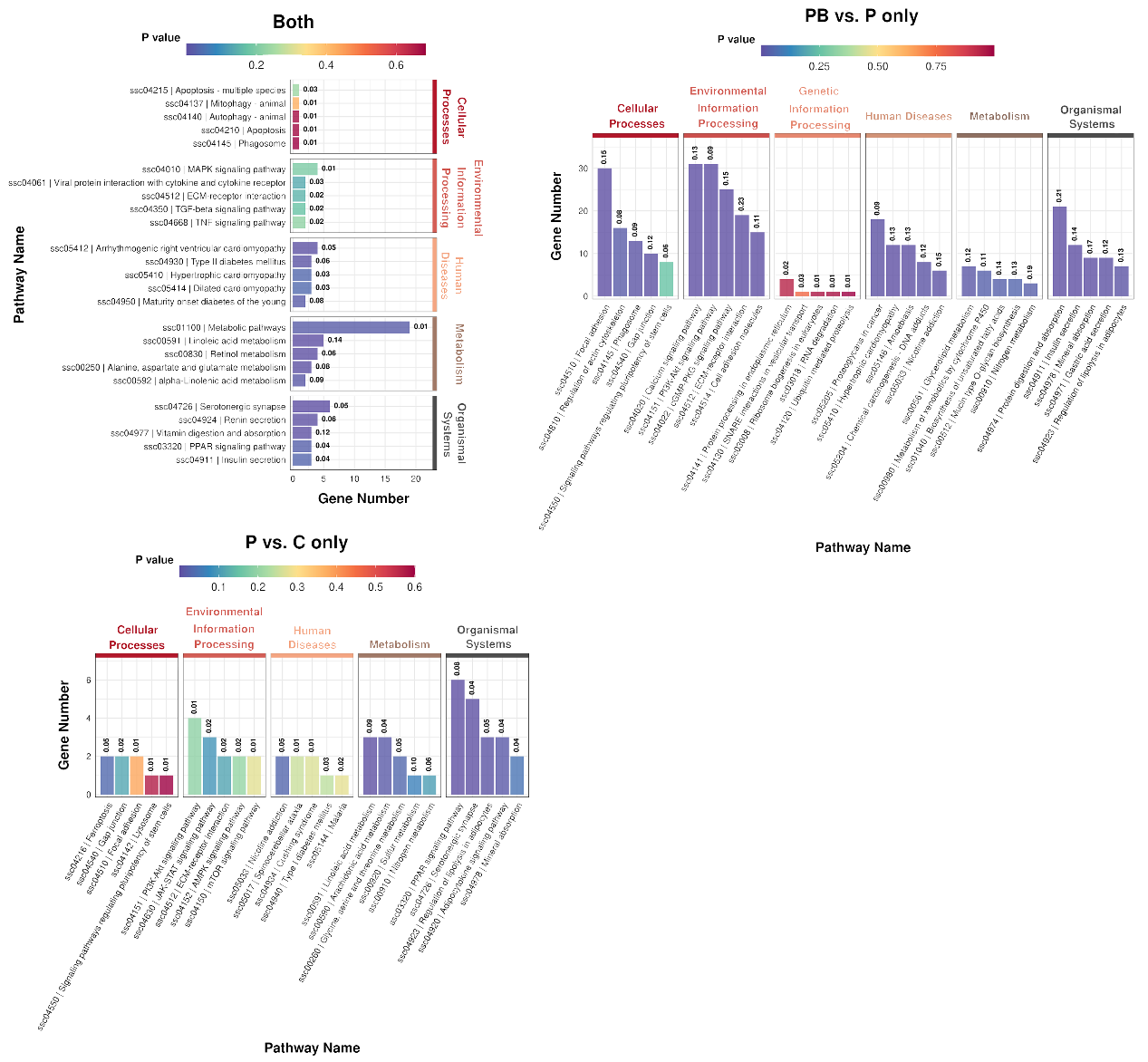


**(C)**

**(B)**

**(A)**

**KEGG analysis based on the genes in subsets of Venn diagram. KEGG analysis for genes differently expressed** in both Pep + Bac vs. Pep and Con vs. Pep (A); Pep vs. Con (B), and Pep + Bac vs. Pep (C). Number above column showing the rich factor of the genes in the column. PB: Pep + Bac; C: Con; P: Pep
