# Supplementary material for: Dual-action peptide KWH2 protects against Salmonella choleraesuis diarrhea in weaned piglets by enhancing intestinal barrier integrity and modulating GSK-3β/Myc signaling
Source: Vet Res. 2026 Mar 17;57:53. doi: 10.1186/s13567-025-01682-x (PMC13104273; doi:10.1186/s13567-025-01682-x)
Supplement: Supplementary file 7 — Additional file 7. Go analysis for genes differently expressed in Pep + Bac versus Pep Only, both in Pep + Bac versus Pep and Con versus Pep, and Con versus Pep only. S: Significant ID number; TS: Total Significant ID Number; B: Background ID Number; ID Number; Rich Factor =S/B. Sig. Sign. P = Significant sign based on P value. [file 13567_2025_1682_MOESM7_ESM.docx]

**Additional file 7 Go analysis for genes differently expressed in Pep + Bac vs. Pep Only, both in Pep + Bac vs. Pep and Con vs. Pep, and Con vs. Pep only.**

**Go analysis for genes differently expressed in Pep + Bac vs. Pep Only.**

S: Significant ID number; TS: Total Significant ID Number; B: Background ID Number; ID Number; Rich Factor =S/B. Sig. Sign. *P* = Significant sign based on *P* value.

**Go analysis for genes differently expressed both in Pep + Bac vs. Pep and Con vs. Pep.**

S: Significant ID number; TS: Total Significant ID Number; B: Background ID Number; ID Number; Rich Factor =S/B. Sig. Sign. *P* = Significant sign based on *P* value.

**Go analysis for genes differently expressed in Con vs. Pep only.**

S: Significant ID number; TS: Total Significant ID Number; B: Background ID Number; ID Number; Rich Factor =S/B. Sig. Sign. *P* = Significant sign based on *P* value.
